# Delimiting Species without Nuclear Monophyly in Madagascar's Mouse Lemurs

**DOI:** 10.1371/journal.pone.0009883

**Published:** 2010-03-31

**Authors:** David W. Weisrock, Rodin M. Rasoloarison, Isabella Fiorentino, José M. Ralison, Steven M. Goodman, Peter M. Kappeler, Anne D. Yoder

**Affiliations:** 1 Department of Biology, Duke University, Durham, North Carolina, United States of America; 2 Department of Behavioural Ecology and Sociobiology, German Primate Centre, Göttingen, Germany; 3 Département de Biologie Animale, Université d'Antananarivo, Antananarivo, Madagascar; 4 Field Museum of Natural History, Chicago, Illinois, United States of America; 5 Vahatra, Antananarivo, Madagascar; 6 Duke Lemur Center, Duke University, Durham, North Carolina, United States of America; Erasmus University Medical Center, Netherlands

## Abstract

**Background:**

Speciation begins when populations become genetically separated through a substantial reduction in gene flow, and it is at this point that a genetically cohesive set of populations attain the sole property of species: the independent evolution of a population-level lineage. The comprehensive delimitation of species within biodiversity hotspots, regardless of their level of divergence, is important for understanding the factors that drive the diversification of biota and for identifying them as targets for conservation. However, delimiting recently diverged species is challenging due to insufficient time for the differential evolution of characters—including morphological differences, reproductive isolation, and gene tree monophyly—that are typically used as evidence for separately evolving lineages.

**Methodology:**

In this study, we assembled multiple lines of evidence from the analysis of mtDNA and nDNA sequence data for the delimitation of a high diversity of cryptically diverged population-level mouse lemur lineages across the island of Madagascar. Our study uses a multi-faceted approach that applies phylogenetic, population genetic, and genealogical analysis for recognizing lineage diversity and presents the most thoroughly sampled species delimitation of mouse lemur ever performed.

**Conclusions:**

The resolution of a large number of geographically defined clades in the mtDNA gene tree provides strong initial evidence for recognizing a high diversity of population-level lineages in mouse lemurs. We find additional support for lineage recognition in the striking concordance between mtDNA clades and patterns of nuclear population structure. Lineages identified using these two sources of evidence also exhibit patterns of population divergence according to genealogical exclusivity estimates. Mouse lemur lineage diversity is reflected in both a geographically fine-scaled pattern of population divergence within established and geographically widespread taxa, as well as newly resolved patterns of micro-endemism revealed through expanded field sampling into previously poorly and well-sampled regions.

## Introduction

Through decades of diverging opinions, at least one component of the species problem – the disagreement over what exactly species are – has found resolution in the consensus view that species are solely defined as separately evolving metapopulation lineages [1], [2], [3]. As such, species exist from the very beginning of their separation and divergence from other lineages and the many available criteria for delimiting these lineages thus mark different points in this process [4], [5]. Despite this reconciliation, evolutionary biologists still have great difficulty in recognizing species in the early stages of divergence due to the limited time for differences to evolve that satisfy most delimitation criteria [5]. Identifying lineages in the early stages of species divergence is, nonetheless, extremely important because the study of these lineages is expected to be the most informative about the speciation process [6].

Until recently, species delimitation methods that provide valid biological evidence for the early stages of lineage divergence have been difficult to properly enumerate. Recent developments in the population genetics of speciation may offer new power to resolve recently diverged lineages, however [7]. Genetic patterns generated by population-level processes operating within diverging lineages are expected to contain the signal of speciation [8], [9], [10] even though divergence is not long enough to generate overt phylogenetic patterns of independent evolution, such as exclusive monophyly at multiple loci [11], [12]. This is, in part, the basis for favoring the use of monophyly in mitochondrial DNA gene trees as evidence for speciation due to a reduced effective population size relative to nuclear loci [13]. This preference relies on the expectation that an increased rate of lineage sorting leads to mtDNA monophyly prior to nuclear monophyly [14]. Even so, accuracy in species delimitation requires the use of more than a single locus [15] and information from multiple loci, ideally in combination with other types of data, are expected to provide a more robust estimate of independently evolving lineages. Methods that summarize population genetic and genealogical patterns across multiple loci are essential for diagnosing these young evolutionary lineages [16], [17], [18].

Lemurs (Lemuriformes: Primates) are a flagship group in the study of the evolutionary and biogeographic mechanisms that have lead to Madagascar's megadiverse biota [19], [20], [21], but face enormous pressure from human-related activity associated with the destruction of their natural forest habitat [22]. A realistic understanding of the species diversity and boundaries of lemurs is therefore fundamental to understanding the evolution of Malagasy biodiversity and in conserving these threatened primates.

In the past decade, the species diversity of mouse lemurs (genus *Microcebus*) has increased more than seven-fold, largely through the analysis of mtDNA sequence data [23], [24], [25], [26], [27], [28], [29]. This rapid increase in species numbers has led to a questioning of the true level of lemur species diversity [30] and debate over whether or not this represents overdiagnosis or a true representation of species diversity. The sole utilization of mtDNA, typical of these recent studies, is problematic in that such data do not address the lack of independence of substitutions among nucleotide positions and ignores the potential discord between gene trees and species trees [31], [32], [33]. For example, the exhibition of female philopatry in mouse lemurs [34], [35] suggests that the maternally inherited mtDNA locus may be strongly biased in such cases.

In this paper, we present the most thoroughly sampled species delimitation study of Malagasy mouse lemurs to date. We present our analyses as a working example of species delimitation where many lineages are morphologically cryptic and recently diverged. These small nocturnal primates also typify the complexities that evolutionary biologists face in assessing diversity in an organismal group that is difficult to study. In the case of mouse lemurs, which are phenotypically very similar, morphological data in most cases cannot be used to separate putative cryptic species, available sample sizes are typically small, both within and among populations, and ecological and behavioral data are often lacking. Instead of viewing these groups as systematically intractable, we argue that the accumulation of substantial genetic data allows us to progress towards a general assessment of species delimitation. Given that lemurs are for the most part forest-dependent, the stakes are often very high, with habitat destruction perhaps erasing species as fast as we can identify and study them [36].

**Figure 1:**
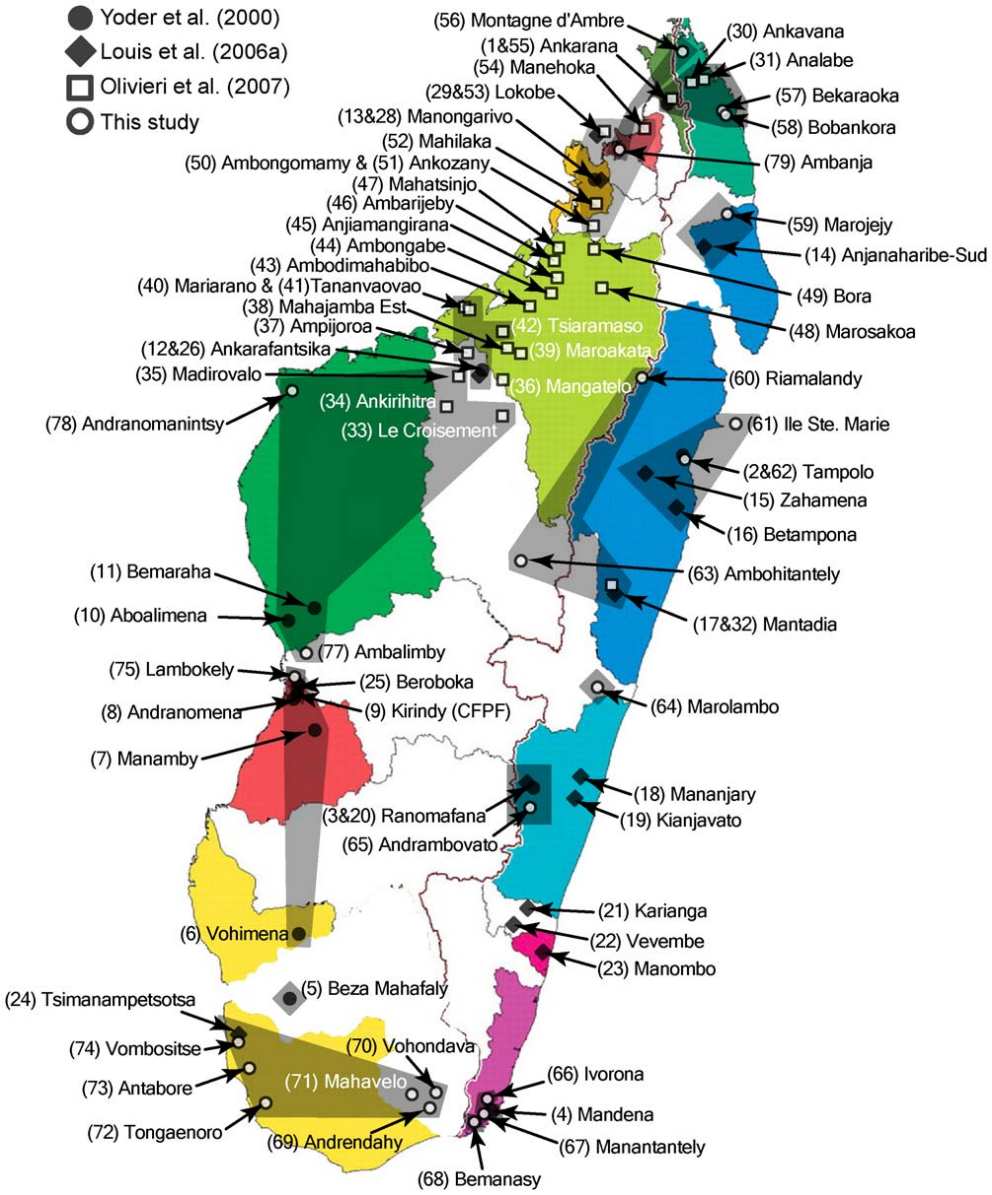
The geographic positions for sampled *Microcebus* localities presented in this study. Numbers in parentheses refer to a detailed listing of localities found in Table S1. Localities marked with an open circle are new to this study and are represented with mtDNA and nDNA data. Localities marked with a filled circle are from Yoder et al. (2000) and Heckman et al. [39] and are represented by mtDNA and nDNA sequence data. Localities marked with filled diamonds and open squares are from Louis et al. [25] and Olivieri et al. [26], respectively, and are represented only by mtDNA data. The island is broken up into regions of micro-endemism (colored sections) and retreat-dispersion (outlined white sections) as defined by Wilmé et al. [21]. Shaded polygons overlay the known distribution of lineages resolved in this study. Localities not encompassed by a shaded polygon are represented by a mtDNA clade that cannot be linked to a nuclear genotypic cluster, limiting a full species delimitation assessment. Due to the extensive overlap of *M. murinus* with other lineages across the western portion of the island, its full range is not depicted.

In the case of the present study, we geographically sampled mouse lemurs from the remaining forested areas across Madagascar, covering virtually all of the island's unique biomes and micro-endemic regions (Fig. 1) [21]. While the number of individuals from any given locality is sometimes reduced, the total sample of 216 individuals (286, with the inclusion of GenBank mtDNA data) represents the effort of numerous field biologists from a time span of more than a decade. As such, it is the most complete synthesis to date, though future sampling efforts will continue to augment our understanding of mouse lemur evolutionary diversity. Although previous studies of mouse lemur evolutionary diversity have included morphological data [28], [37], here we focus primarily on genetic data sampled from mtDNA and four independent nuclear loci. We assess patterns of monophyly across independently reconstructed gene trees to identify lineages that exhibit genealogical exclusivity, an expected pattern for lineages with long durations of divergence [11], [12], [15]. However, the larger focus of this study is on the delimitation of population-level lineages that have a relatively recent history of divergence. We use a combined data approach to identify distinct nuclear genetic clusters using analyses of population structure [18], [38]. We test hypotheses of diverging lineages identified through the mtDNA gene tree and nuclear clustering with recently developed statistics that quantify the degree of exclusive ancestry in an assemblage of gene trees [17]. As such, this study offers an opportunity to fully explore the strengths, as well as the limitations, of genetic data for the interpretation of species boundaries. In conclusion, we aim to show that a well-developed multi-locus genetic data set that is analyzed appropriately can yield penetrating insights into the history and reproductive boundaries both within and among the evolutionary lineages that we infer to be species.

## Materials and Methods

### Organismal sampling

MtDNA sequence data were analyzed from 286 individual mouse lemurs distributed across Madagascar (Fig. 1). 102 of these represent new material from 23 previously unsampled localities distributed throughout Madagascar. MtDNA sequence data from the remaining individuals comes from previous work [25], [26], [37]. Nuclear sequence data were analyzed from a total of 216 mouse lemurs. As with the mtDNA data, 102 of these are new material from the 23 previously unsampled localities. Nuclear sequence data from the remaining samples are those of Heckman et al. [39], which are from the same localities presented in the mtDNA work of Yoder et al. (2000). Full details regarding the number of individuals per locality, locality names, and geographic coordinate data can be found in Table S1. The nuclear sequence data of Heckman et al. [39] required editing for the identification of previously unidentified heterozygous nucleotide positions, and for the refinement of allele phases, and are deposited in GenBank with new accession numbers. Outgroup sequences for phylogenetic analysis were obtained from GenBank for single individuals of each of the following species: *Cheirogaleus crossleyi*, *C. major*, *C. medius*, and *Mirza coquereli*. GenBank accession numbers for all outgroup individuals and for the mtDNA data of Louis et al. [25] and Olivieri et al. [26] can be found in Table S2. All new sequence data and sequence data from the studies of Yoder et al. [37] and Heckman et al. [39] are available in GenBank with the following accession numbers: mtDNA *cox2* (GU326974-GU327160), mtDNA *cob* (GU327161-GU327362), *adora3* (GU230899-GU231330), *eno* (GU231331-GU231716), *fga* (GU231717-GU232130), and *vwf* (GU232131-GU232490).

### Genetic Sampling and Data Collection

MtDNA sequence data from two separate regions, *cox2* and *cob*, were generated using PCR and direct sequencing with the PCR primers L7553/H8320 [40] and L14724/H15915 [41], respectively. Nuclear DNA sequence data were generated from four independent loci (*adora3*, *fga*, *eno*, and *vwf*). These are the same loci used in Heckman et al. [39] and PCR primer information are found within that reference except for those used in the amplification of *adora3*, which are from Horvath et al. [42]. PCR was performed in volumes of 20 µl using 2 µl template DNA (approximately 50-150 ng DNA), 25 µM each dNTP, 1 µM each primer, and 0.625U Taq polymerase in a standard 1x reaction buffer. Typical mtDNA amplification conditions were carried out with an initial 94°C denaturation for 2 min, followed by 35 cycles of 30 s at 94°C, 30 s at 50°C and 45 s at 68°C. A final 7 minutes extension was performed at 72°C. See Horvath et al. (2008) for specific annealing conditions for each nuclear locus. PCR products were directly sequenced using both forward and reverse PCR primers and BigDye® Terminator v3.1 (Applied Biosystems, Foster City, CA). Prior to sequencing, 9 µl PCR product was treated with 1.5U exonuclease I and 0.3U shrimp alkaline phosphatase. Cycle sequencing was performed in a total volume of 5 µL including 1 µl Exo/SAP treated product, 2 µM primer, 0.5 µl BDv3.1 and water to 5 µl. Cycle sequencing conditions were carried out for 25 cycles: 95°C for 10 sec, 55°C for 5 sec, 60°C for 2 min and a final hold at 10°C. Fluorescent traces were analyzed using an ABI 3730xl DNA Analyzer (Applied Biosystems, Foster City, CA). Most nuclear PCR products that generated sequence exhibiting polymorphic sites or length heterogeneity were cloned using a Topo® TA cloning kit (Invitrogen, Carlsbad, CA), and for each cloned PCR, eight colonies were sequenced to identify alleles. Haplotypes for some heterozygous sequences were phased using an algorithmic approach implemented in PHASE v2.1 [43]. Within each nuclear locus we estimated the minimum number of recombination events [44] using DnaSP v4.0 [45]. We tested for departure from neutral evolution within individual loci using Fu and Li's F* statistics [46].

### Phylogenetic Reconstruction

Bayesian phylogenetic analysis was performed on single-gene haplotype data sets and on single-gene full nuclear data sets including all individuals and their respective gene copies using MrBayes v3.1.2 [47]. Evolutionary models for each locus were assessed for the haplotype data sets using Akaike Information Criteria in MrModeltest v2.3 [48]. MtDNA data for individual mouse lemurs were concatenated and analyzed in a two-partition framework with model parameters estimated separately for the *cox2* and *cytb* genes. Nuclear gene data sets were analyzed as a single partition. Four Markov chains were used with the default temperature parameter of 0.2. Default priors were used in all analyses and random trees were used to start each Markov chain. Chains were run for 10 million generations with topology and model parameter estimates sampled every 1000 generations. The first five million generations were discarded as burn-in yielding a posterior distribution of 5000 sampled trees. Mean log likelihood (lnL), branch lengths and topologies were compared across four replicate analyses to insure that a stable posterior distribution was reached. Sampled trees from the posterior distributions of replicate analyses were pooled and parsed with MrBayes to construct a majority rule consensus tree and to calculate posterior probabilities (PPs) of all resulting branches. We assessed nuclear monophyly of terminal clades resolved in the mtDNA haplotype tree and for previously described taxa by filtering the nuclear Bayesian posterior distributions for trees that meet that constraint of monophyly for specified groups. Tree filtering was performed in PAUP* version 4.0 [49].

### Bayesian Structure Analysis

Analysis of population structure was assessed using a genotype matrix of the nuclear loci in STRUCTURE v2.2 [50], [51]. We ran a series of analyses under models assuming a specific number of populations (*K*), with a range of *K* from 2 to 26. In each iteration, individuals were assigned probabilistically to a cluster based on their multilocus genotype. All analyses used one million MCMC generations to estimate the posterior distribution following a burnin period of one million generations. Our model incorporated the possibility that some individuals may have mixed population ancestry and the possibility that allele frequencies are correlated among populations due to migration or shared ancestry [51]. For each *K*, the log (ln) probability of the data (X) was estimated [ln Pr(X|*K*)] and used to calculate the posterior probability (PP) of *K* under the assumption of a uniform prior. We also calculated Δ*K* [52], which is based on the rate of change in ln Pr(X|*K*) between successive *K* values. These two measures often identify different optimal measures of *K*, and Δ*K* may favor smaller values of *K* that represent basal levels of hierarchical structure in systems that substantially deviate from an island model [52]. We visualized the optimal *K* STRUCTURE plot using Microsoft Excel. Membership coefficients for individuals with posterior probabilities less than 0.05 were disregarded and proportionally assigned to the other cluster assignment coefficients.

### Genealogical Tests of Population Divergence

We assessed the level of genealogical divergence in our nuclear gene trees for hypothesized lineages identified in the mtDNA gene tree and nuclear STRUCTURE analysis using the genealogical sorting index (*gsi*) [17]. For pre-defined groups in a gene tree, the *gsi* is a standardized measure of the degree to which they exhibit exclusive ancestry. The *gsi* statistic ranges from 1 (monophyly) to 0 (a complete lack of genealogical divergence with other groups). A major benefit of the *gsi* statistic for species delimitation is the ability to assess its statistical significance through the randomization of group labels across the tips in a gene tree. Consequently, hypothesized lineages can be tested against a null hypothesis of no divergence, as measured by coalescent patterns in their gene trees. For each individual nuclear locus, we calculated the *gsi* for 100 trees randomly sampled from the combined Bayesian posterior distribution of trees. These 100 individual *gsi* measurements were then used together with equal weight to calculate an ensemble *gsi* statistic (*gsi_T_*) for each locus. Thus, g*si_T_* measurements serve as a summary of the genealogical exclusivity across the Bayesian posterior distribution of trees for a given locus. In addition to the single-locus assessments of genealogical exclusivity, we also calculated *gsi_T_* for the set of majority-rule consensus trees of all four nuclear loci. The significance of all *gsi* and *gsi_T_* statistics was assessed using 1000 randomization permutations. All analyses were performed using the Genealogical Sorting Index web server (www.genealogicalsorting.org).

## Results

Information regarding size, level of variability, molecular evolutionary models, and likelihood values from phylogenetic analyses for all mtDNA and nDNA genes can be found in Table 1. No evidence was found for recombination within any of the four nuclear loci. Furthermore, no locus yielded a significant signature of a departure from neutrality.

**Table 1:** Information for loci used in this study.

Locus	Size (bp)	Variable Sites	No. of Haplotypes	Favored Model^a^	Mean Bayesian lnL (haplotype data)	Mean Bayesian lnL (full data)	ML lnL (full data)
mtDNA (*cox2*)	684	176	169^b^	HKY+I+G	−13360.0^c^ (−13400.0, −13320.0)	—	—
mtDNA (*cytb*)	1184	396	169^b^	HKY+I+G	—	—	—
*adora3*	384	34	38	HKY+G	−1104.6 (−1118.3, −1093.08	−1223.7 (−1244.3, −1204.9)	−852.9
*eno*	913	150	156	HKY+I+G	−4557.6 (−4582.1, −4531.5)	−4717.33 (−4754.1, −4684.4)	−3915.7
*fga*	632	74	82	HKY+I+G	−2232.9 (−2251.07, −2215.7)	−2433.1 (−2460.1, −2406.9)	−1953.0
*vwf*	824	117	140	HKY+I+G	−3980.1 (−4007.5, −3953.3)	−4164.8 (−4230.8, −4099.0)	−3379.7

a Model selected according to the Akaike Information Criterion in the program MrModelTest.

b Haplotype number includes sequence data from GenBank and is calculated for the concatenated set of mtDNA data.

c Scores are calculated for the concatenated mtDNA data.

### MtDNA Gene Tree

**Figure 2:**
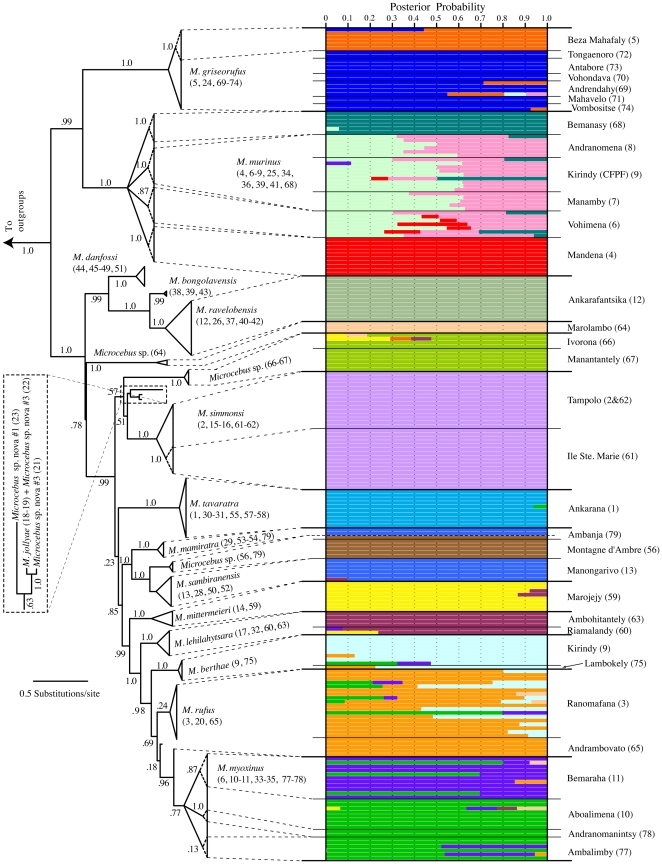
Correspondence between clades in the mtDNA gene tree and nuclear Structure clusters. The mtDNA gene tree results from Bayesian analysis of concatenated *cox2* and *cytb* sequence. It is presented as a maximum credibility topology with branch lengths averaged across the posterior distribution. Numbers on branches represent posterior probabilities. Relationships within terminal clades are collapsed for ease of presentation and clades are labeled according to present species designations. Locality numbers are given in parentheses and correspond to Fig. 1 and Table S1. Clades are mapped to corresponding clusters in the nuclear STRUCTURE plot. Each cluster is designated by a different color with horizontal bars representing individuals and the proportion of a bar assigned to a single color representing the posterior probability that an individual is assigned to that cluster. This can also be interpreted as the percentage of an individuals genome that is derived from that particular genetic cluster. Localities from which individuals are sampled from are given along the right side of the plot. MtDNA clades not mapped to the assignment plot represent individuals for which corresponding nDNA data is not available. The colors in the Bayesian assignment plot do not correspond to colored areas of micro-endemism in Fig. 1.

The mtDNA haplotype tree resolved the majority of previously described species as clades (Fig. 2, Table 2; full mtDNA gene tree details are presented in Figure S1) with just two exceptions: *M*. *rufus* mtDNA haplotypes are paraphyletic and *M*. *jollyae* and *Microcebus* sp. nova 3 [25] share a mtDNA haplotype. MtDNA variation is highly structured across the island with numerous sampling localities within currently recognized species exhibiting monophyly (dashed-line clades in Fig. 2). In addition, some sets of localities within described species are reconstructed as geographically paraphyletic groups (e.g. within *M. griseorufus* and *M. myoxinus*). Three novel mtDNA clades are resolved based on new field sampling: (1) haplotypes sampled from Marolambo (locality 64), (2) haplotypes sampled from the localities of Ivorona and Manantantely (localities 66 and 67), and (3) haplotypes sampled from Ambanja and Montagne d'Ambre (Localities 56 and 79). This latter clade may represent *M. arnholdi*, a recently described species from Montagne d'Ambre that was diagnosed using patterns of mtDNA divergence [29]. However, that study used different mtDNA gene regions than those used here, preventing a direct link with the lineage resolved in our mtDNA gene tree. Therefore, we treat the Ambanja+Montagne d'Ambre lineage resolved here as an unnamed lineage with the understanding that it may represent *M. arnholdi*.

**Table 2:** Probability of monophyly in the single-gene Bayesian posterior distributions and nuclear cluster assignments for *Microcebus* species and populations.

Lineage	Posterior probability for monophyly					Bayesian assignment cluster(s) w/average individual membership coefficients	MtDNA group + genotypic cluster
	mtDNA	*adora3*	*eno*	*fga*	*vwf*		
*M. griseorufus*	**1.0**	0	0.99	0	0.0004	1 (0.28), 2 (0.71), **1+2 (1.0)**	**Yes**
*M. murinus* – sensu lato	**1.0**	0	0.04	0	0	3 (0.17), 4 (0.29), 5 (0.28), 6 (0.25), **3+4+5+6 (0.99)**	-
– Bemanasy	**1.0**	0	0	0	0	**3 (0.99)**, 4 (0.01)	**Yes**
– And/Kir/Manam/Vohi	0.0^a^	0	0	0	0	3 (0.05), 4 (0.47), 5 (0.45), **4+5 (0.92)**, 6 (0.03)	**Yes** a
– Mandena	**1.0**	0	0	0	0	**6 (1.0)**	**Yes**
*M. danfossi*	**1.0**	No Data	No Data	No Data	No Data	No Data	-
*M. bongolavensis*	**0.99**	No Data	No Data	No Data	No Data	No Data	-
*M. ravelobensis*	**1.0**	0.9781	1.0	1.0	0.9914	**7 (1.0)**	**Yes**
*Microcebus* sp. – Marolambo	**1.0**	0	1.0	0.576	0.205	**8 (1.0)**	**Yes**
*Microcebus* sp. – Ivorona/Manantantely	**1.0**	0	0	0.9754	0	1 (0.01), 8 (0.01), **9 (0.93)**, 14 (0.04), 15 (0.01)	**Yes**
*M. jollyae*	0.0	No Data	No Data	No Data	No Data	No Data	-
*M. simmonsi*	**1.0**	0.9957	1.0	1.0	0.9914	**10 (1.0)**	**Yes**
*M. tavaratra*	**1.0**	0.9318	1.0	0	0.16	**11 (0.99)**, 19 (0.01)	**Yes**
*M. mamiratra*	**1.0**	0.001	0	0	0	12 (1.0)	No
*Microcebus* sp. Ambanja/Montagne d'Ambre	**1.0**	0	0.301	0	0	**13 (1.0)**	**Yes**
*M. sambiranensis*	**1.0**	0.149	0	0	0	**12 (.98)**, 15 (0.02)	**Yes**
M. mittermeieri	**1.0**	0	0	0	0	**14 (0.97)**, 15 (0.03)	**Yes**
*M. lehilahytsara*	**1.0**	0	0	0	0	14 (0.04), **15 (0.95)**, 18 (0.01)	**Yes**
*M. berthae*	**1.0**	0	0	0	0	**16 (0.91),** 17 (0.04), 18 (0.02), 19 (0.03)	**Yes** c
*M. rufus*	0^b^	0	0	0	0	8 (0.01), 16 (0.12), 17 (0.78), 18 (0.02), 19 (0.07)	**Yes** c
*M. myoxinus*	**0.77**	0	0	0	0	8 (0.01), 14 (∼0), 15 (∼0), 17 (0.01), 18 (0.36), 19 (0.62), **18+19 (0.98)**	**Yes** c
Total number of diagnosed lineages =							**16**

For definitions of the locality acronyms within *M. murinus* see Table S1.

a The populations of Andranomena+Kirindy(CFPF)+ Mamamby and the population of Vohimena each form separate mtDNA clades.

b *Microcebus rufus* is paraphyletic with respect to *M. myoxinus*.

c *Microcebus berthae* and *M. myoxinus* both are placed into distinct nuclear clusters that are also present in greater than 5% of the average cluster assignments of *M. rufus*. We diagnose these as three distinct clusters based on their geographic distributions (see discussion).

### Nuclear Gene Trees

Bayesian phylogenetic analysis of haplotype data for the individual nuclear genes results in majority rule consensus trees that resolve only a subset of the clades resolved in the mtDNA gene tree (Figures S2, S3, S4, S5). Filtering of trees from the full-data Bayesian posterior distributions reveals that few of the mtDNA-based clades exhibit monophyly with posterior probabilities greater than 0.05 (Table 2). The exceptions to this are *M. griseorufus*, *M. sambiranensis*, *Microcebus* sp. (Ambanja+Montagne d'Ambre), and *Microcebus* sp. (Ivorona-+Manantantely), all of which are monophyletic in one nuclear gene tree, *M. mamiratra*, which is monophyletic in two nuclear gene trees, *M. tavaratra* and *Microcebus* sp. (Marolambo), both of which are monophyletic in three nuclear gene trees, and *M. ravelobensis* and *M. simmonsi*, which exhibit monophyly in all four nuclear gene trees (Table 2). Finally, construction of a strict consensus tree from the ML nuclear gene trees does not yield reciprocal monophyly for additional groups that are not present in the mtDNA gene tree (results not shown), but instead yields reciprocal monophyly for just two species, *M. ravelobensis* and *M. simmonsi*.

### Nuclear Population Structure

Plots of the estimated log probability of the data [log Pr(X|*K*)] for replicated STRUCTURE analyses reveal a general pattern of a plateau or decrease in values above a *K* = 19 (Figure S6). Four replicate analyses yield a posterior probability of 1.0 for a *K* = 19 (Figure S6). The remaining replicate analyses had posterior probabilities of 1.0 for *K*s of 20, 23, 24, and 26. In contrast, calculations of Δ*K* produce a peak at *K* = 2 (Figure S6). This result likely stems from the identification of a basal level of hierarchical structure in the data [52]. The plateau in patterns of the log Pr(X|*K*) around a *K* = 19, and the high posterior probabilities for *K*≥19, suggest an overall higher population cluster number. A plot of individual membership coefficients for *K* = 19 reveals a high number of population clusters with average individual membership coefficients (i.e. posterior probabilities) greater than 0.9 (Figure 2, Table 2). STRUCTURE plots for *K*>19 do not yield additional clusters with high membership coefficients for more exclusive sets of populations or individuals, but instead further divide already admixed sets of individuals (e.g. populations within *M. murinus*) into additional admixed cluster assignments. Therefore, we place our focus on *K* = 19 as an estimate of the upper level of population clustering.

There is strong concordance between identified nuclear clusters and terminal mtDNA clades (Fig. 2). Of the 12 mtDNA clades representing described species, and for which we have corresponding nuclear data, 11 map to one or more nuclear genotypic clusters characteristic to that clade. Many populations within described species that exhibit mtDNA monophyly also map to distinct nuclear clusters with high individual membership coefficients (Table 2). For example, all individuals sampled from the *M. murinus* population of Mandena (locality 4) have a monophyletic assemblage of mtDNA haplotypes and an assignment to a single nuclear STRUCTURE cluster with average membership coefficients of 1.0. The three novel mtDNA clades from the populations of Marolambo, Ivorona+Manantantely, and Ambanja+Montagne d'Ambre also each map to their own respective nuclear clusters with average individual membership coefficients of 1.0, 0.93, and 1.0, respectively. In total, using corresponding patterns of nuclear STRUCTURE clustering and mtDNA monophyly, we diagnose a total of 16 hypothesized lineages of *Microcebus* (Table 2). These criteria focus on the assignment of mtDNA clade-specific localities or sets of localities to one or more characteristic nuclear clusters with limited signs of admixture (i.e. average cluster membership coefficients ≤5% for other localities).

In contrast to these patterns, individuals within the mtDNA haplotype clade that correspond to the recently described species, *M. mamiratra*, have nuclear STRUCTURE assignments identical to individuals of *M. sambiranensis* (Fig. 2, Table 2). Similar patterns are also seen for some localities within described species that exhibit mtDNA monophyly, but do not have high membership coefficients to distinct nuclear clusters (e.g. the locality of Vohimena within *M. murinus* and the locality of Ambalimby within *M. myoxinus*; Fig. 2, Table 2).

### Nuclear Genealogical Sorting

Overall, there is considerable variation in measures of genealogical divergence across hypothesized lineages, across gene trees within lineages, and across the Bayesian posterior distribution of individual gene trees (Table 3). Nonetheless, significant measures of exclusive ancestry are estimated at all of these levels of organization with just two exceptions (Table 3): (1) the *adora3* Bayesian posterior distribution contains >5 individual trees in which the *gsi* for *M. lehilahytsara* is not significant; however, the *adora3 gsi_T_* for *M. lehilahytsara* significantly rejects the null hypothesis of no divergence, and (2) the *adora3* Bayesian posterior distribution contains >5 individual trees in which the *gsi* for the undescribed lineage (*Microcebus* sp.) from Marolambo is not significant. However, in this latter case the overall *adora3 gsi_T_* measurement is not significant. *Gsi_T_* values for most lineages are lower for the *adora3* locus than the other three nuclear loci, a result consistent with the overall lower level of genetic variation in the *adora3* data set (Table 1).

**Table 3:** Ensemble genealogical sorting indices (*gsi_T_*) for the combined set of majority-rule consensus trees from all four nuclear loci and for the Bayesian posterior distributions of each individual nuclear locus.

Lineage	All loci *gsi_T_*	*adora3 gsi_T_* (min-max)	*eno gsi_T_* (min-max)	*fib gsi_T_* (min-max)	*vwf gsi_T_* (min-max)
*M. berthae*	**0.158**	**0.185 (0.141–0.269)**	**0.573 (0.377–0.843)**	**0.259 (0.207–0.343)**	**0.303 (0.222–0.412)**
*M. griseorufus*	**0.487**	**0.414 (0.257–0.519)**	**0.729 (0.683–0.814)**	**0.673 (0.664–0.717)**	**0.48 (0.359–0.576)**
*M. lehilahytsara*	**0.069**	**0.119** (0.074–0.187)^a^	**0.243 (0.155–0.529)**	**0.407 (0.27–0.538)**	**0.226 (0.149–0.344)**
*M. mittermeieri*	**0.142**	**0.141 (0.103–0.211)**	**0.384 (0.255–0.643)**	**0.361 (0.179–0.639)**	**0.512 (0.304–0.739)**
*M. murinus* (Bemanasy)	**0.157**	**0.224 (0.153–0.327)**	**0.204 (0.168–0.247)**	**0.2 (0.141–0.28)**	**0.323 (0.246–0.487)**
*M. murinus* (Mandena)	**0.357**	**0.314 (0.255–0.377)**	**0.768 (0.765–0.862)**	**0.323 (0.246–0.45)**	**0.474 (0.4–0.571)**
*M. murinus* (remaining pops)	**0.406**	**0.469 (0.395–0.571)**	**0.892 (0.842–0.926)**	**0.36 (0.279–0.506)**	**0.45 (0.317–0.57)**
*M. myoxinus*	**0.416**	**0.341 (0.311–0.392)**	**0.522 (0.441–0.631)**	**0.429 (0.368–0.489)**	**0.736 (0.672–0.793)**
*M. ravelobensis*	**1.0**	**1.0 (0.956–1.0)**	**1.0 (1.0–1.0)**	**0.995 (0.727–1.0)**	**1.0 (1.0–1.0)**
*M. rufus*	**0.326**	**0.302 (0.25–0.357)**	**0.533 (0.45–0.67)**	**0.431 (0.369–0.5)**	**0.7 (0.62–0.829)**
*M. sambiranensis*	**0.491**	**0.913 (0.334–1.0)**	**0.407 (0.293–0.605)**	**0.344 (0.203–0.465)**	**0.756 (0.559–0.935)**
*M. simmonsi*	**1.0**	**1.0 (0.981–1.0)**	**1.0 (1.0–1.0)**	**1.0 (1.0–1.0)**	**0.999 (0.944–1.0)**
*M. tavaratra*	**0.724**	**0.981 (0.495–1.0)**	**1.0 (1.0–1.0)**	**0.359 (0.194–0.511)**	**0.895 (0.761–1.0)**
*Microcebus* sp. (Ambanja/Montagne d'Ambre)	**0.270**	**0.259 (0.126–0.538)**	**0.892 (0.725–1.0)**	**0.516 (0.296–0.78)**	**0.383 (0.183–0.536)**
*Microcebus* sp. (Ivorona/Manantantely)	**0.478**	**0.141 (0.125–0.23)**	**0.669 (0.589–0.8)**	**1.0 (1.0–1.0)**	**0.383 (0.187–0.566)**
*Microcebus* sp. (Marolambo)	**0.713**	0.097 (0.062–0.183)^a^	**1.0 (1.0–1.0)**	**0.966 (0.831–1.0)**	**0.861 (0.71–1.0)**

a >5 of the 100 trees sampled from the Bayesian posterior distribution had *gsi* values with p>0.05.

b *Microcebus rufus* is not diagnosed as a diverging lineage according to the criteria used in this study, but is included here based on patterns of population differentiation from *M. berthae* and *M. myoxinus* and due to its delimitation based on morphological and ecological traits [37].

The minimum and maximum *gsi* for individual trees within the Bayesian posterior distributions are given in parentheses.

## Discussion

### Species delimitation in mouse lemurs

Speciation begins when populations become genetically separated through a substantial reduction in gene flow, either through vicariance, or through selection and adaptation, and it is at this point that a genetically cohesive set of populations attain the sole property of species: the independent evolution of a population-level lineage [1], [3]. The delimitation of lineages in these early stages of speciation is important because their study is most likely to yield insights into the mechanisms that drove their formation [6]. The comprehensive delimitation of all diverging lineages within hotspots of biodiversity, regardless of their level of divergence, is important for understanding the factors driving the diversification of biota [e.g. 21] and guiding biologically realistic conservation action [53]. However, delimiting recently diverged lineages is challenging due to insufficient time for the differential evolution of characters – including morphological differences, reproductive isolation, and monophyly in gene trees – that are typically used as evidence for separately evolving lineages [5]. The most likely patterns to evolve over short time scales of divergence are the population genetic patterns of differentiation. The reduction of gene flow among populations allows genetic drift to operate independently within cohesive sets of populations yielding distinctive patterns in allele frequencies [54] and in gene trees [9], [55]. The application of species delimitation criteria that identify lineages exhibiting the population genetic patterns of cohesion through gene flow [18], [38] and genealogical patterns of divergence [16], [17] can thus have significant benefits over criteria that are more often limited to identifying lineages that are well into the process of divergence.

In this study, we assembled multiple lines of evidence from the analysis of mtDNA and nDNA sequence data for the delimitation of numerous cryptically diverged lineages of mouse lemurs across the island of Madagascar. This evidence included one of the more standard phylogeographic components of lineage diagnosis: the resolution of clades in a mtDNA gene tree. However, there is an overall lack of corresponding nuclear monophyly for most mtDNA clades. Only two population-level lineages, the previously described *M. ravelobensis* and *M. simmonsi*, are delimited via monophyly in all mtDNA and nDNA gene trees (Table 2), and just nine lineages are delimited when this criterion was relaxed to monophyly in mtDNA and at least one nuclear gene tree. Interestingly, these monophyly criteria fail to delimit many lineages previously described as species on the basis of clear and concordant patterns of mtDNA, morphological, and ecological differentiation [28], [37], [56]. In contrast, STRUCTURE analysis of the nuclear data clustered individuals into groups that strongly align with mtDNA clades (Fig. 2). Furthermore, despite a lack of nuclear monophyly for most of these population groupings, estimates of their *gsi* significantly favor independent histories of lineage divergence (Table 3). Within the framework of the general lineage concept of species [1], [3], which clarifies that new species arise at the very beginning of lineage divergence from an ancestor, corroborating lines of evidence that indicate the independent divergence of a lineage provide substantial support for its delimitation as a species [5]. We, therefore, present our study as a working example of a comprehensive approach for delimitating recently diverged species and elaborate on the individual components of this approach below.

The resolution of a large number of geographically defined clades in the mtDNA gene tree (Fig. 2) provided a strong suggestion for a high diversity of mouse lemur lineages. Many mtDNA clades correspond to groups resolved in previous mtDNA-based studies [24], [25], [26], [37]. This is in large part due to our inclusion of GenBank mtDNA data from these previous studies, which facilitated the placement of much of our nuclear results within the context of previous species delimitation work. Novel to this study, however, was the resolution of additional mtDNA clades resolved on the basis of two different sampling regimes. First, expanded field sampling led to the resolution of mtDNA clades within described species that are specific to one or a few sampling localities. For example, within the *M. murinus* mtDNA clade the localities of Bemanasy and Mandena were each resolved as monophyletic sets of haplotypes (Fig. 2). This fine scale pattern of mtDNA differentiation was also seen in paraphyletic groupings within described species. For example, both *M. griseorufus* and *M. myoxinus* contained paraphyletic groups with respect to other single-locality monophyletic groupings (Table 2). Both of these phylogenetic patterns represent a potentially fine-scale level of geographic genetic divergence not previously considered within mouse lemurs. Second, we also resolved three novel and relatively divergent mtDNA clades (each labeled *Microcebus* sp. in Fig. 2), each of which corresponds to new localities that had not previously been sampled. These patterns indicate that the upper limit of our understanding of mouse lemur lineage diversity may also be constrained by our ability to comprehensively sample across Madagascar.

Much of the recent flurry of mouse lemur species descriptions has been based primarily on patterns in mtDNA gene trees. While the sorting of mtDNA variation may, on average, track lineage divergence more rapidly than nDNA [14], there are important reasons for not relying on mtDNA as a sole source of evidence in species delimitation, including the potential to over diagnose mtDNA lineages influenced by selection [15] or sex-biased dispersal [57]. Accordingly, the strongest genetic evidence for speciation comes from evaluating patterns of variation across multiple loci [15], [58]. We find additional evidence for mouse lemur lineage divergence in the striking degree of concordance between mtDNA clades and patterns of nuclear population genetic structure. Localities or sets of localities that are resolved as a clade in the mtDNA gene tree are predominantly assigned to a characteristic nuclear genotypic cluster with high average assignment probabilities (0.9–1.0) in the STRUCTURE assignment plot (Fig. 2, Table 2). There are only limited signs of admixture between some population clusters, a pattern consistent with mtDNA evidence for a lack of gene flow between groups. In contrast to the evolution of nuclear monophyly, which can require long durations of time [(∼4-7N generations for just 50% of the nuclear genome's gene trees [15]], the evolution of allele frequency differences and genome-wide patterns of Hardy-Weinberg and linkage equilibrium within lineages is expected to occur more rapidly. Population assignment analyses based on these parameters may serve as useful methods for delimiting the early stages of lineage divergence [18], [38].

One potential criticism of our interpretation of the STRUCTURE results is that these patterns simply represent the geographic structuring of intraspecific variation. For example, STRUCTURE analysis places human population genetic variation into geographically-defined clusters [59]. A major benefit to the use of STRUCTURE analysis is its ability to identify individuals with admixed genomic profiles that arise through gene flow between previously isolated population clusters [51]. Human populations are undoubtedly cohesive through gene flow, as evidenced, in part, by signatures of admixture in their STRUCTURE profiles. However, this does not negate STRUCTURE's ability to identify cluster patterns that reflect *histories* of isolation and divergence. In fact, the models implemented in STRUCTURE specifically allow for the reconstruction of ancestral clusters even if most individuals are largely composed of admixed genotypes [51]. Consequently, we contend that, within natural systems, populations characterized by distinct genotypic clusters with limited signs of admixture exhibit at least one layer of evidence for lineage divergence.

To further test the hypothesis that these sets of mouse lemur populations have an underlying history of lineage divergence, we quantified the magnitude of their genealogical divergence and tested whether or not genealogical patterns within lineages were significantly different from those expected under a history of no divergence. Accompanying the speciation process is an expected transition, driven by genetic drift, in the gene genealogies of a diverging lineage from polyphyletic sets of ancestral gene copies to monophyletic sets of unique gene copies [12], [33]. Consequently, characteristic topological patterns are expected to evolve in the gene trees of a diverging lineage long before the evolution of monophyly, and these patterns can be used to distinguish independent and recently diverged sets of populations [16], [17]. All mouse lemur lineages delimited via mtDNA monophyly and nuclear clustering have significant patterns of genealogical exclusivity in their nuclear gene trees, as measured by *gsi* values for the Bayesian posterior distributions of individual gene trees, and by the ensemble *gsi_T_*, which integrates patterns across loci (Table 3). These results clarify our interpretations of the nuclear STRUCTURE clusters by providing a genealogical perspective of their underlying history. Despite the fact that the majority of nuclear clusters do not represent monophyletic groups in their component nuclear gene trees, they nonetheless have genealogical patterns consistent with a history of lineage divergence. *Gsi_T_* values across loci indicate a broad range in the degree of genealogical exclusivity across loci (0.069–1.0). However, because the progression to monophyly is governed by both time and effective population size [33], [55], it is difficult to place lineages on a relative temporal scale of divergence based on their overall *Gsi_T_*. Instead, the resolution of lineages with significant gene tree-wide *Gsi_T_* values provides a simple, but quantitative indication of divergence for many lineages that have not been diverging long enough to have evolved concordant patterns of monophyly across the majority of their gene trees.

**Figure 3:**
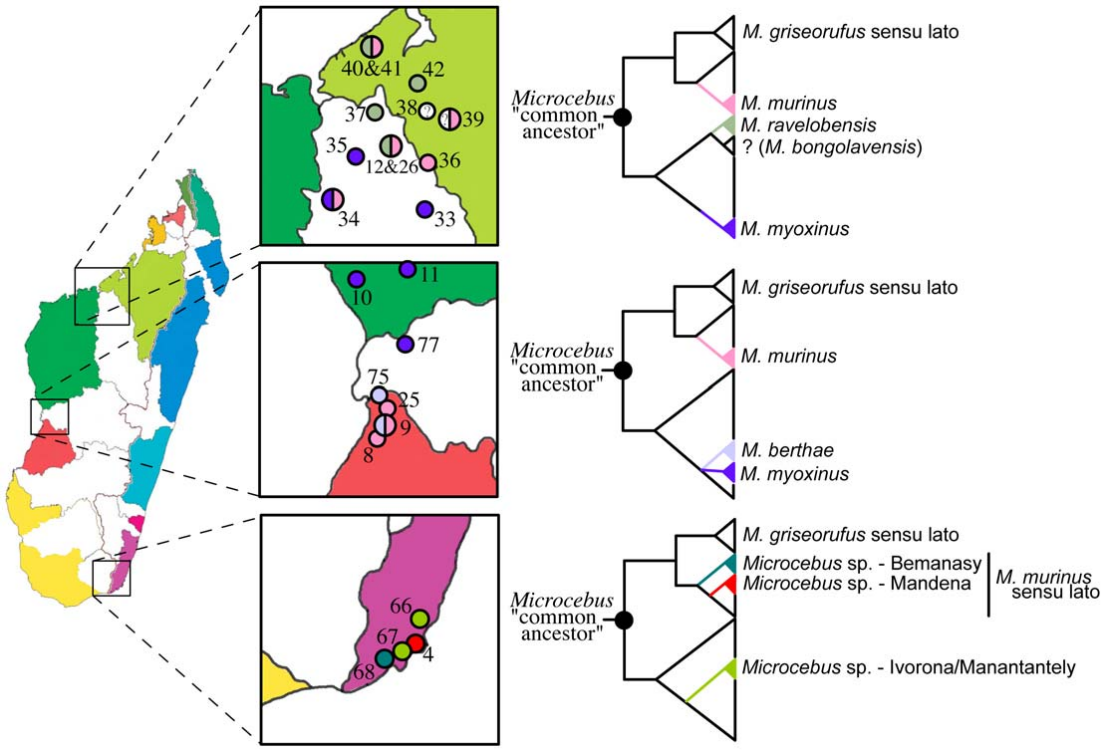
Phylogenetic descriptions of areas of sympatry and fine-scale allopatry among *Microcebus* lineages. Colored sections in the maps of Madagascar signify areas of micro-endemism and white sections signify retreat dispersion regions [21]. All known regions of sympatry in mouse lemurs involve populations of *M*. *murinus* sensu lato. The top two outlined boxes highlight regions of sympatry involving *M*. *murinus* and at least three other species (*M*. *bongolavensis* could not be assessed in this study), each of which shares a most recent common ancestor with *M. murinus* at the root of most gene trees. However, at least one case of sympatry is known that involves *M. murinus* and its sister lineage, *M. griseorufus*, in the Berenty Reserve in southern Madagascar [not sampled in this study and not depicted here [69]. As further support for the speciation model of Wilmé et al. (2006), areas of sympatry appear to be clustered in or near retreat dispersion regions, which are proposed to have influenced the expansion of species ranges during the Quaternary. In contrast, the bottom outlined box highlights lineage distributions within a single region of micro-endemism in the southeast.

Based on this total set of evidence, we diagnose 16 population-level lineages of mouse lemurs (Tables 2, 3). Within the framework of the metapopulation lineage concept of species [1], [3], all of these lineages are recognized as species, regardless of their relative level of divergence. In contrast, species criteria that place greater emphases on more explicit properties of species (e.g. reproductive isolation or gene tree monophyly) would yield a substantially lower number of delimited and recognized species. Without placing emphasis on the number of species formally recognized by our results, we highlight the strong evidence for considerable lineage formation within *Microcebus*, including lineages with genetic patterns indicative of long durations of independent divergence, as well as those with patterns suggestive of a shallower depth of divergence. In total, these results are counter to a recently proposed argument that the escalation in lemur species diversity is largely due to oversplitting of geographically localized variants that are merely components of larger cohesive lineages [30]. Instead, our estimates of a high lineage diversity of mouse lemurs is reflected in both a geographically fine-scale pattern of population divergence within established and geographically widespread species, as well as newly resolved patterns of micro-endemism revealed through expanded field sampling into previously poorly and well-sampled regions. Fine-scale patterns of lineage divergence are particularly pronounced within *M*. *murinus* in the southeastern populations of Bemanasy and Mandena, which are genetically diverged and distinct from each other and from western *M*. *murinus* populations. The geographic scale of divergence between these two southeastern lineages within *M*. *murinus* across a pronounced bioclimatic ecotone [60] is striking, with a physical separation of just 27 km (Fig. 3), but genetic patterns that seem to exclude any sign of genetic exchange. Moreover, new regions of micro-endemic lineages are revealed. From the newly-sampled Marolambo population (locality 64) on the central eastern side of the island, a novel lineage was discovered that displays no evidence of gene flow with *M*. *lehilahytsara* or *M*. *simmonsi* to the north, or *M*. *rufus* to the south (Fig. 2). In the well-sampled regions of Ivorona and Manantantely (localities 66 and 67) in the extreme southeast, we observe sympatric distributions of three distinct lineages (Fig. 3) that show no evidence of gene flow, despite the fact that these populations are separated by no more than 10 km. Yet, another novel lineage is detected in the northern populations of Ambanja (locality 79) and Montagne d'Ambre (locality 56). This latter lineage may equate with a recently described species from Montagne d'Ambre, *M. arnholdi*, which was described on the basis of mtDNA divergence from other northern populations [29]. However, the lack of overlapping mtDNA regions across studies limits a direct connection between the lineage diagnosed here and *M. arnholdi*. Overall, these patterns of lineage divergence suggest that mouse lemur speciation can occur at a broad range of geographic levels, and indeed, indicates the probability of even more as-yet-undescribed lineage diversity. A number of additional species have been described in recent years that we cannot address with the nuclear data in this study [25], [26], [27], [29] and future work will be needed to assess the validity of these described taxa as independent lineages.

In contrast, the analytical approach and criteria used here also identified patterns reflecting the signal of population divergence, but without straightforward evidence for independent evolution. This is best exemplified with a set of three previously described species: *M*. *berthae* and *M*. *myoxinus* in the western part of the island, and *M*. *rufus* in the east. Each is distinct in the mtDNA gene tree (*M*. *rufus* is paraphyletic), is largely comprised of a characteristic nuclear cluster in STRUCTURE analyses (Fig. 2), and has significant *gsi* estimates. However, while individuals within *M*. *berthae* and *M*. *myoxinus* are predominantly assigned to distinct nuclear clusters, the *M*. *rufus* population of Ranomafana has an average of 12% of its overall membership coefficients attributed to the *M*. *berthae* cluster and 9% attributed to *M. myoxinus* clusters (Table 2). As a comparison, no other species diagnosed in this study had an average membership coefficient of >5% from any other species' nuclear cluster. These patterns are suggestive of gene flow into the range of *M*. *rufus*. In such cases, determining the evolutionary independence of these potential lineages will require additional sources of information. In this particular case, geography is particularly informative: the western lineages (*M. berthae* and *M. myoxinus*) are highly genetically distinct from each other (Fig. 2, Table 2), but are extremely close geographically (Figs. 1, 3). In contrast, both are separated from *M. rufus* by a high elevation north-south mountain system (Fig. 1). These geographic patterns indicate that if gene flow is the cause of the slightly mixed cluster assignment for *M. rufus*, then it is historical in nature, and currently all three lineages (*M*. *berthae*, *M*. *myoxinus*, and *M*. *rufus*) are genetically isolated. As such, these patterns suggest that all three are diverging population-level lineages.

Finally, we not only identify sets of independently evolving lineages with this approach, but also identify cohesive sets of populations, particularly in situations where mtDNA distinctiveness would suggest divergence. This is best exemplified with the recently described *M*. *mamiratra* from northwest Madagascar [24], [26]. Our results find some individuals from Ambanja (locality 79) to contain mtDNA haplotypes placed within the *M*. *mamiratra* clade, which nonetheless have a nuclear structure profile indistinguishable from individuals of *M*. *sambiranensis* (Fig. 2). We therefore propose that the diagnosis of *M. mamiratra* is based on insufficient structure in mtDNA variation and should not be recognized. Similar patterns exist within the currently recognized taxa *M*. *murinus*, *M*. *myoxinus*, and *M*. *simmonsi*. Within each of these taxa, subsets of populations exhibit monophyly in the mtDNA gene tree (Fig. 2), suggestive of lineage divergence; yet, these subpopulations also exhibit nuclear STRUCTURE profiles that indicate an admixed background of nuclear gene flow and cohesion with other populations (Fig. 2). These contrasting patterns between the mtDNA and nuclear genetic data may be related to the propensity for female philopatry in lemurs [34], [35] and highlight the potential pitfalls of relying solely on mtDNA gene tree patterns in the delimitation of mouse lemur species.

### Mouse lemur evolution

What do these results have to say about the mechanism of speciation in *Microcebus*, and about lemur diversification in general? The isolated geographic distributions of most mouse lemur species (Fig. 2) indicate a strong role for allopatric speciation. Furthermore, while we are currently limited in our ability to infer a robust species tree and divergence time estimates for mouse lemur lineages, the predominant lack of reciprocal monophyly across nuclear gene trees (Figures S2, S3, S4, S5) suggests relatively recent divergence times for most. These two inferences align with a recent model for the origin of micro-endemic regions in Madagascar, which places emphasis on reduced precipitation during the Quaternary and formation of forest refugia in lower elevation river catchments around the periphery of the island [21]. A number of mouse lemur lineages lie within, or mostly within, these areas of micro-endemism (Fig. 1), indicating that this model may account for diversification at some level. Additional diversification models developed for Madagascar, including the roles of climate [61], rivers [19], and mountains [62] also remain to be tested with a robust estimate of the mouse lemur species tree [63].

Also of interest is the paralleled high level of recently recognized species diversity in other nocturnal lemur genera (e.g. *Avahi*, *Lepilemur*), suggesting a link between nocturnal activity and unrealized population genetic structure. An important behavioral mechanism in nocturnal lemurs is acoustic signaling [64], a necessary form of communication in the dark. Mouse lemur species in particular have evolved distinctly different male advertisement calls [65], providing a potential mechanism for premating isolation that can limit admixture among populations that have previously experienced allopatric divergence. Contrastingly, however, a study of the closely related and nocturnal dwarf lemurs (*Cheirogaleus*) using the same markers and some of the methods applied here finds a disproportionately low level of taxonomic diversity [3 diagnosable species as compared to 7 proposed in recent years [66]], despite having a similar island-wide distribution [67].

We provide here the most comprehensive species delimitation study of lemurs ever performed and are the first to integrate gene tree analysis, Bayesian STRUCTURE analysis, and quantifications of genealogical divergence in the identification of population-level lineages. While our results confirm the high lineage diversity results of previous mtDNA-based studies, this should not serve as justification for limiting future studies to a single marker in a DNA-barcoding protocol. Instead, our results regarding the lack of corresponding nuclear evidence of divergence for some resolved mtDNA lineages and the under appreciation of fine-scale geographic patterns of lineage divergence not previously highlighted in mtDNA studies emphasizes the need for a more thorough approach utilizing appropriate genetic data and analytical methods. The approach outlined here may also serve as a general model for lineage diagnosis of other groups with a predominant pattern of cryptic diversity. For example, recent surveys of Malagasy frogs found an additional 129 mtDNA lineages that either lack clear morphological or acoustic differences, or that have not yet been studied with independent data that can be used to test their distinctiveness [68]. Testing these lineages with the population genetic and genealogical framework used here may provide an ideal alternative to further field and museum-based assessments of species delimitation.

## Supporting Information

Table S1 Locality and sampling information for all *Microcebus* localities used in this study.
(0.19 MB DOC)

Table S2 GenBank accession numbers for all outgroup sequence data and the *Microcebus* mtDNA sequence data of Louis et al. [24] and Olivieri et al. [25].
(0.12 MB DOC)

Figure S1 MtDNA gene tree. The tree results from Bayesian phylogenetic analysis of the mtDNA haplotype data set and is presented as the maximum credible topology with branch lengths averaged across the posterior distribution (Mean -lnL = 1.33×10^4^, Std. Dev. = 7.76). Tip labels include a species name if the haplotype was sampled from an individual identified to a species in a previous study. Haplotypes recovered from newly sampled individuals are indicated with the locality name.
(1.84 MB TIF)

Figure S2 *adora3* gene tree. The tree results from Bayesian phylogenetic analysis of the *adora3* haplotype data set and is presented as the maximum credible topology with branch lengths averaged across the posterior distribution (Mean -lnL = 1104.67, 95% HPD = 1118.35-1093.08). Tip labels include a species name if the haplotype was sampled from an individual identified to a species in a previous study. Haplotypes recovered from newly sampled individuals are indicated with the locality name.
(1.54 MB TIF)

Figure S3 *eno* gene tree. The tree results from Bayesian phylogenetic analysis of the *eno* haplotype data set and is presented as the maximum credible topology with branch lengths averaged across the posterior distribution (Mean -lnL = 4557.6, 95%HPD = 4582.15-4531.58). Tip labels include a species name if the haplotype was sampled from an individual identified to a species in a previous study. Haplotypes recovered from newly sampled individuals are indicated with the locality name.
(1.74 MB TIF)

Figure S4 *fga* gene tree. The tree results from Bayesian phylogenetic analysis of the *fga* haplotype data set and is presented as the maximum credible topology with branch lengths averaged across the posterior distribution (Mean -lnL = 2232.92, 95%HPD = 2251.07-2215.76). Tip labels include a species name if the haplotype was sampled from an individual identified to a species in a previous study. Haplotypes recovered from newly sampled individuals are indicated with the locality name.
(1.84 MB TIF)

Figure S5 *vwf* gene tree. The tree results from Bayesian phylogenetic analysis of the *vwf* haplotype data set and is presented as the maximum credible topology with branch lengths averaged across the posterior distribution (Mean -lnL = 3980.14, 95%HPD = 4007.55-3953.37). Tip labels include a species name if the haplotype was sampled from an individual identified to a species in a previous study. Haplotypes recovered from newly sampled individuals are indicated with the locality name.
(1.81 MB TIF)

Figure S6 Plots of calculations for various K values in STRUCTURE analysis of the nuclear data. (a) The log probability of the data for K = 10 to 26. Colored lines represent replicate STRUCTURE analyses. (b) Posterior probabilities for K = 10 to 26 for replicate STRUCTURE analyses. Different dashed lines represent replicate analyses, many of which have the same posterior probability. (c) ΔK values for K = 2 to 25.
(1.55 MB TIF)
