# Supplementary material for: Delimiting Species without Nuclear Monophyly in Madagascar's Mouse Lemurs
Source: PLoS One. 2010 Mar 31;5(3):e9883. doi: 10.1371/journal.pone.0009883 (PMC2847600; doi:10.1371/journal.pone.0009883)
Supplement: Table S1 — Locality and sampling information for all Microcebus localities used in this study. (0.19 MB DOC) [file pone.0009883.s001.doc]

| Study | Locality # | Locality Name | Species | Coordinates | mtDNA | *adora3* | *fga* | *eno* | *vwf* |
| --- | --- | --- | --- | --- | --- | --- | --- | --- | --- |
| **Yoder et al. (2000)** | 1 | Ankarana | *M. tavaratra* | -13º 03' 00", +49º 03' 00" | 9 | 9 | 9 | 9 | 9 |
|  | 2 | Tampolo | *M. simmonsi* | -17º 16' 60", +49º 25' 00" | 2 | 2 | 2 | 2 | 2 |
|  | 3 | Ranomafana (Ifanadiana) | *M. rufus* | -21º 15' 00", +47º 27' 00" | 19 | 18 | 17 | 13 | 15 |
|  | 4 | Mandena | *M. murinus* | -24º 58' 00", +47º 01' 00" | 10 | 10 | 11 | 8 | 10 |
|  | 5 | Beza Mahafaly | *M. griseorufus* | -23º 40' 00", +44º 37' 00" | 6 | 6 | 6 | 6 | 6 |
|  | 6 | Vohimena [Vohi] | *M. murinus* | -22º 40' 00", +44º 49' 00" | 6 | 7 | 6 | 5 | 5 |
|  | 7 | Manamby [Manam] | *M. murinus* | -20º 25' 60", +44º 49' 60" | 5 | 5 | 5 | 5 | 4 |
|  | 8 | Andranomena [And] | *M. murinus* | -20º 09' 00", +44º 33' 00" | 5 | 6 | 6 | 6 | 6 |
|  | 9 | Kirindy (CFPF) | *M. berthae* | -20º 03' 00", +44º 39' 00" | 6 | 8 | 8 | 8 | 6 |
|  | 9 | Kirindy (CFPF) [Kir] | *M. murinus* | -20º 03' 00", +44º 39' 00" | 4 | 9 | 9 | 8 | 2 |
|  | 10 | Aboalimena | *M. myoxinus* | -19º 15' 00", +44º 27' 00" | 7 | 8 | 8 | 8 | 8 |
|  | 11 | Bemaraha | *M. myoxinus* | -19º 06' 00", +44º 47' 60" | 11 | 10 | 11 | 11 | 10 |
|  | 12 | Ankarafantsika | *M. ravelobensis* | -16º 19' 60", +46º 46' 60" | 11 | 12 | 12 | 12 | 10 |
|  | 13 | Manongarivo | *M. sambiranensis* | -14º 01' 00", +48º 15' 00" | 6 | 6 | 6 | 5 | 6 |
|  |  |  |  |  |  |  |  |  |  |
| **Louis et al.**  **(2006a)** | 14 | Anjanaharibe-Sud | *M. mittermeiri* | -14º 47' 27", +49º 27' 53" | 6 | - | - | - | - |
|  | 15 | Zahamena | *M. simmonsi* | -17º 29' 13", +48º 44' 30" | 4 | - | - | - | - |
|  | 16 | Betampona | *M. simmonsi* | -17º 55' 52", +49º 12' 12" | 5 | - | - | - | - |
|  | 17 | Mantadia | Louis sp. nova 2 | -18º 48' 29", +48º 25' 29" | 1 | - | - | - | - |
|  | 18 | Mananjary | *M. jollyae* | -21º 07' 30", +48º 01' 03" | 1 | - | - | - | - |
|  | 19 | Kianjavato | *M. jollyae* | -21º 22' 42", +47º 52' 06" | 2 | - | - | - | - |
|  | 20 | Ranomafana (Ifanadiana) | *M. rufus* | -21º 15' 00", +47º 27' 00" | 5 | - | - | - | - |
|  | 21 | Karianga | Louis sp. nova 3 | -22º 25' 20", +47º 22' 12" | 1 | - | - | - | - |
|  | 22 | Vevembe | Louis sp. nova 3 | -22º 47' 22", +47º 11' 02" | 2 | - | - | - | - |
|  | 23 | Manombo | Louis sp. nova 1 | -23º 01' 42", +47º 43' 50" | 1 | - | - | - | - |
|  | 24 | Tsimanampetsotsa | *M. griseorufus* | -24º 05' 10", +43º 45' 09" | 3 | - | - | - | - |
|  | 25 | Beroboka | *M. murinus* | -19º 58' 36", +44º 39' 59" | 5 | - | - | - | - |
|  | 26 | Ankarafantsika | *M. ravelobensis* | -16º 21' 43", +46º 45' 60" | 5 | - | - | - | - |
|  | 27 | Antafondro | Louis sp. nova 5 | -14º 02' 56", +48º 13' 18" | - | - | - | - | - |
|  | 28 | Manongarivo | *M. sambiranensis* | -14º 01' 15", +48º 16' 12" | 1 | - | - | - | - |
|  | 29 | Lokobe | *M. mamiratra* | -13º 24' 26", +48º 18' 10" | 4 | - | - | - | - |
|  |  |  |  |  |  |  |  |  |  |
| **Olivieri et al. (2007)** | 30 | Ankavana | *M. tavaratra* | -12º 46' 33", +49º 22' 16" | 2 | - | - | - | - |
|  | 31 | Analabe | *M. tavaratra* | -12º 45' 14", +49º 30' 04" | 1 | - | - | - | - |
|  | 32 | Mantadia | *M. lehilahytsara* | -18º 47' , +48º 25' | 2 | - | - | - | - |
|  | 33 | Le Croisement |  | -16º 51' 24", +47º 01' 30" | 1 | - | - | - | - |
|  | 34 | Ankirihitra | *M. murinus/M. myoxinus* | not available | 2 | - | - | - | - |
|  | 35 | Madirovalo | *M. myoxinus* | -16º 22' 47", +46º 29' 02" | 1 | - | - | - | - |
|  | 36 | Mangatelo | *M. murinus* | -16º 24' 30", +46º 58' 19" | 1 | - | - | - | - |
|  | 37 | Ampijoroa | *M. ravelobensis* | not available | - | - | - | - | - |
|  | 38 | Mahajamba Est | *M. bongolavensis* | -15º 59' 34"; +47º 10' 15" | 1 | - | - | - | - |
|  | 39 | Maroakata | *M. bongolavensis* | -16º 04' 57", +47º 18' 05" | 2 | - | - | - | - |
|  | 40 | Mariarano | *M. ravelobensis* | -15º 28' 50", +46º 41' 19" | 1 | - | - | - | - |
|  | 41 | Tananvaovao | *M. ravelobensis* | -15º 28' 16", +46º 39' 59" | 1 | - | - | - | - |
|  | 42 | Tsiaramaso | *M. ravelobensis* | -15º 47' 57", +47º 07' 21" | 1 | - | - | - | - |
|  | 43 | Ambodimahabibo | *M. bongolavensis* | -15º 29' 54", +47º 28' 47" | 1 | - | - | - | - |
|  | 44 | Ambongabe | *M. danfossi* | -15º 19' 38", +47º 40' 44" | 1 | - | - | - | - |
|  | 45 | Anjiamangirana | *M. danfossi* | -15º 09' 25", +47º 44' 06" | 1 | - | - | - | - |
|  | 46 | Ambarijeby |  | -14º 56' 29", +47º 42' 44" | 1 | - | - | - | - |
|  | 47 | Mahatsinjo | *M. danfossi* | -14º 47' 40", +47º 47' 01" | 1 | - | - | - | - |
|  | 48 | Marasakoa | *M. danfossi* | -15º 15' 42", +48º 17' 57" | 1 | - | - | - | - |
|  | 49 | Bora | *M. danfossi* | -14º 51' 42", +48º 12' 26" | 1 | - | - | - | - |
|  | 50 | Ambongomamy | *M. sambiranensis* | -14º 29' 43", +48º 12' 35" | 1 | - | - | - | - |
|  | 51 | Ankozany | *M. danfossi* | -14º 31' 43", +48º 12' 27" | 1 | - | - | - | - |
|  | 52 | Mahilaka | *M. sambiranensis* | -14º 17' 12", +48º 13' 38" | 2 | - | - | - | - |
|  | 53 | Lokobe | *M. lokobensis* | -13º 23' 24", +48º 20' 31" | 1 | - | - | - | - |
|  | 54 | Manehoko | *M. lokobensis* | -13º 25' 49", +48º 47' 51" | 2 | - | - | - | - |
|  | 55 | Ankarana | *M. tavaratra* | -12º 58' 05", +49º 08' 19" | 1 | - | - | - | - |
|  |  |  |  |  |  |  |  |  |  |
| **This Study** | 56 | Montagne d’Ambre |  | -12º 28' 30", +49º 13' 06" | 5 | 5 | 5 | 5 | 5 |
|  | 57 | Bekaraoka |  | -13º 09' 57", +49º 43' 03" | 1 | 0 | 0 | 0 | 0 |
|  | 58 | Bobankora |  | -13º 13' 33", +49º 45' 04" | 1 | 0 | 0 | 0 | 0 |
|  | 59 | Marojejy |  | -14º 28' 02", +49º 50' 21" | 8 | 8 | 8 | 8 | 8 |
|  | 60 | Riamalandy |  | -16º 17' 06", +48º 48' 54" | 2 | 2 | 2 | 2 | 2 |
|  | 61 | Ile Ste. Marie |  | -15º 55' 22", +49º 52' 29" | 16 | 16 | 16 | 16 | 16 |
|  | 62 | Tampolo |  | -17º 17' 13", +49º 24' 32" | 13 | 13 | 13 | 13 | 13 |
|  | 63 | Ambohitantely |  | -18º 28' 34", +47º 16' 26" | 4 | 4 | 4 | 4 | 4 |
|  | 64 | Marolambo |  | -15º 55' 22", +49º 52' 29" | 3 | 3 | 3 | 3 | 3 |
|  | 65 | Andrambovato |  | -21º 29' 45", +47º 24' 06" | 5 | 5 | 5 | 5 | 5 |
|  | 66 | Ivorona |  | -24º 49' 25", +46º 56' 55" | 4 | 4 | 4 | 3 | 3 |
|  | 67 | Manantantely |  | -24º 59' 17", +46º 55' 20" | 6 | 6 | 6 | 6 | 5 |
|  | 68 | Bemanasy |  | -25º 05' 07", +46º 46' 31" | 6 | 6 | 6 | 5 | 6 |
|  | 69 | Andrendahy |  | -24º 52' 06", +46º 23' 54" | 4 | 4 | 4 | 4 | 4 |
|  | 70 | Vohondava |  | -24º 41' 12", +46º 27' 12" | 2 | 2 | 2 | 2 | 2 |
|  | 71 | Mahavelo |  | -24º 45' 30", +46º 09' 06" | 2 | 2 | 2 | 2 | 2 |
|  | 72 | Tongaenoro |  | -24º 44' 12", +44º 1' 48" | 2 | 2 | 2 | 2 | 2 |
|  | 73 | Antabore |  | -24º 23' 54", +43º 50' 48" | 4 | 4 | 4 | 4 | 4 |
|  | 74 | Vombositse |  | -24º 11' 18", +43º 45' 54" | 2 | 2 | 2 | 2 | 2 |
|  | 75 | Lambokely |  | -19º 52' 12", +44º 38' 42" | 1 | 1 | 1 | 1 | 1 |
|  | 77 | Ambalimby |  | -19º 36' 42", +44º 44' 36" | 6 | 6 | 6 | 6 | 6 |
|  | 78 | Andranomanitsy |  | -16º 31' 12", +44º 29' 12" | 2 | 2 | 2 | 2 | 2 |
|  | 79 | Ambanja |  | -13º 42' 11", +48º 30' 16" | 3 | 3 | 3 | 3 | 3 |
|  |  |  |  |  | 286 | 216 | 216 | 204 | 197 |
