# Supplementary material for: Delimiting Species without Nuclear Monophyly in Madagascar's Mouse Lemurs
Source: PLoS One. 2010 Mar 31;5(3):e9883. doi: 10.1371/journal.pone.0009883 (PMC2847600; doi:10.1371/journal.pone.0009883)
Supplement: Table S2 — GenBank accession numbers for all outgroup sequence data and the Microcebus mtDNA sequence data of Louis et al. [24] and Olivieri et al. [25]. (0.12 MB DOC) [file pone.0009883.s002.doc]

| Locality # | Locality Name | Species | *cox2* (mtDNA) | *cob*  (mtDNA) | *adora3* | *fga* | *eno* | *vwf* |
| --- | --- | --- | --- | --- | --- | --- | --- | --- |
|  |  | *Cheirogaleus crossleyi* | EU825498 | EU825354 | EU825313- EU825314 | EU825433- EU825434 | - | EU825577- EU825578 |
|  |  | *Cheirogaleus major* | EU825480 | EU825336 | EU825257- EU825258 | EU825397- EU825398 | GU361973 | EU825541- EU825542 |
|  |  | *Cheirogaleus medius* | EU825477 | EU825333 | EU825239- EU825240 | EU825391- EU825392 | - | EU825535- EU825536 |
|  |  | *Mirza coquereli* | U53571 | AY321460 | EU342234 | EU342261 | GU361972 | AY434036 |
| **Louis et al. (2006)** |  |  |  |  |  |  |  |  |
| 14 | Anjanaharibe-Sud | *M. mittermeiri* | AY569182, AY569192 | - | - | - | - | - |
| 15 | Zahamena | *M. simmonsi* | AY515551, AY515552, AY515553, AY569203 | - | - | - | - | - |
| 16 | Betampona | *M. simmonsi* | AY515549, AY515550, AY569188, AY569189, AY569190 | - | - | - | - | - |
| 17 | Mantadia | Louis sp. nova 2 | AY569179 | - | - | - | - | - |
| 18 | Mananjary | *M. jollyae* | AY569184 | - | - | - | - | - |
| 19 | Kianjavato | *M. jollyae* | AY569183, AY569193 | - | - | - | - | - |
| 20 | Ranomafana (Ifanadiana) | *M. rufus* | AY515547, AY515548, AY569198, AY569199, AY569200 | - | - | - | - | - |
| 21 | Karianga | Louis sp. nova 3 | AY515543 | - | - | - | - | - |
| 22 | Vevembe | Louis sp. nova 3 | AY569201, AY569202 | - | - | - | - | - |
| 23 | Manombo | Louis sp. nova 1 | AY515556 | - | - | - | - | - |
| 24 | Tsimanampetsotsa | *M. griseorufus* | AY515545, AY515546, AY569197 | - | - | - | - | - |
| 25 | Beroboka | *M. murinus* | AY515554, AY515555, AY569194, AY569195, AY569196 | - | - | - | - | - |
| 26 | Ankarafantsika | *M. ravelobensis* | AY515544, AY596178, AY569185, AY569186, AY569187 | - | - | - | - | - |
| 28 | Manongarivo | *M. sambiranensis* | AY569191 | - | - | - | - | - |
| **Olivieri et al. (2007)** |  |  |  |  |  |  |  |  |
| 30 | Ankavana | *M. tavaratra* | EF065240, EF065241 | EF065199, EF065212 | - | - | - | - |
| 31 | Analabe | *M. tavaratra* | EF065242 | EF065206 | - | - | - | - |
| 32 | Mantadia | *M. lehilahytsara* | EF065255, EF065243 | EF065200, EF065181 | - | - | - | - |
| 33 | Le Croisement |  | EF065247 | EF065188 | - | - | - | - |
| 34 | Ankirihitra | *M. murinus/M. myoxinus* | EF065265, EF065266 | EF065189, EF065183 | - | - | - | - |
| 35 | Madirovalo | *M. myoxinus* | EF065252 | EF065182 | - | - | - | - |
| 36 | Mangatelo | *M. murinus* | EF065257 | EF065184 | - | - | - | - |
| 38 | Mahajamba Est | *M. bongolavensis* | EF065254 | EF065185 | - | - | - | - |
| 39 | Maroakata | *M. bongolavensis* | EF065262, EF065263 | EF065186, EF065190 | - | - | - | - |
| 40 | Mariarano | *M. ravelobensis* | EF065251 | EF065197 | - | - | - | - |
| 41 | Tananvaovao | *M. ravelobensis* | EF065248, EF065269 | EF065191, EF065196 | - | - | - | - |
| 42 | Tsiaramaso | *M. ravelobensis* | EF065259 | EF065198 | - | - | - | - |
| 43 | Ambodimahabibo | *M. bongolavensis* | EF065256 | EF065195 | - | - | - | - |
| 44 | Ambongabe | *M. danfossi* | EF065260 | EF065194 | - | - | - | - |
| 45 | Anjiamangirana | *M. danfossi* | EF065267 | EF065192 | - | - | - | - |
| 46 | Ambarijeby |  | EF065253 | EF065205 | - | - | - | - |
| 47 | Mahatsinjo | *M. danfossi* | EF065268 | EF065204 | - | - | - | - |
| 48 | Marosakoa | *M. danfossi* | EF065258 | EF065193 | - | - | - | - |
| 49 | Bora | *M. danfossi* | EF065246 | EF065203 | - | - | - | - |
| 50 | Ambongomamy | *M. sambiranensis* | EF065264 | EF065209 | - | - | - | - |
| 51 | Ankozany | *M. danfossi* | EF065244 | EF065208 | - | - | - | - |
| 52 | Mahilaka | *M. sambiranensis* | EF065249, EF065271 | EF065180, EF065202 | - | - | - | - |
| 53 | Lokobe | *M. mamiratra* | EF065245 | EF065201 | - | - | - | - |
| 54 | Manehoko | *M. mamiratra* | EF065250, EF065270 | EF065207, EF065211 | - | - | - | - |
| 55 | Ankarana | *M. tavaratra* | EF065239 | EF065210 | - | - | - | - |
