# Supplementary figures and images for: Delimiting Species without Nuclear Monophyly in Madagascar's Mouse Lemurs
Source: PLoS One. 2010 Mar 31;5(3):e9883. doi: 10.1371/journal.pone.0009883 (PMC2847600; doi:10.1371/journal.pone.0009883)

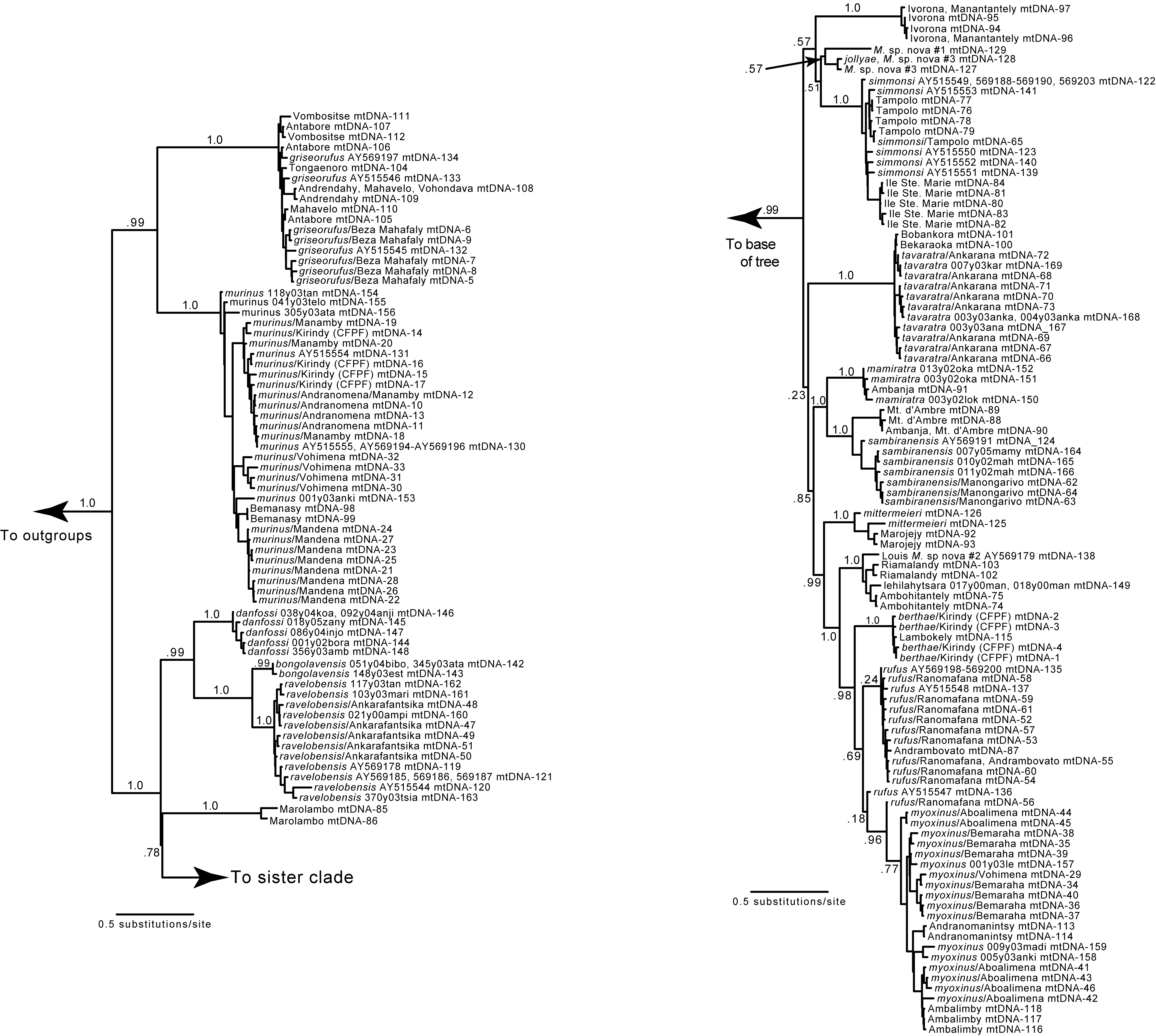

Supplement: Figure S1 — MtDNA gene tree. The tree results from Bayesian phylogenetic analysis of the mtDNA haplotype data set and is presented as the maximum credible topology with branch lengths averaged across the posterior distribution (Mean -lnL = 1.33×10^4^, Std. Dev. = 7.76). Tip labels include a species name if the haplotype was sampled from an individual identified to a species in a previous study. Haplotypes recovered from newly sampled individuals are indicated with the locality name. (1.84 MB TIF) [file pone.0009883.s003.tif]

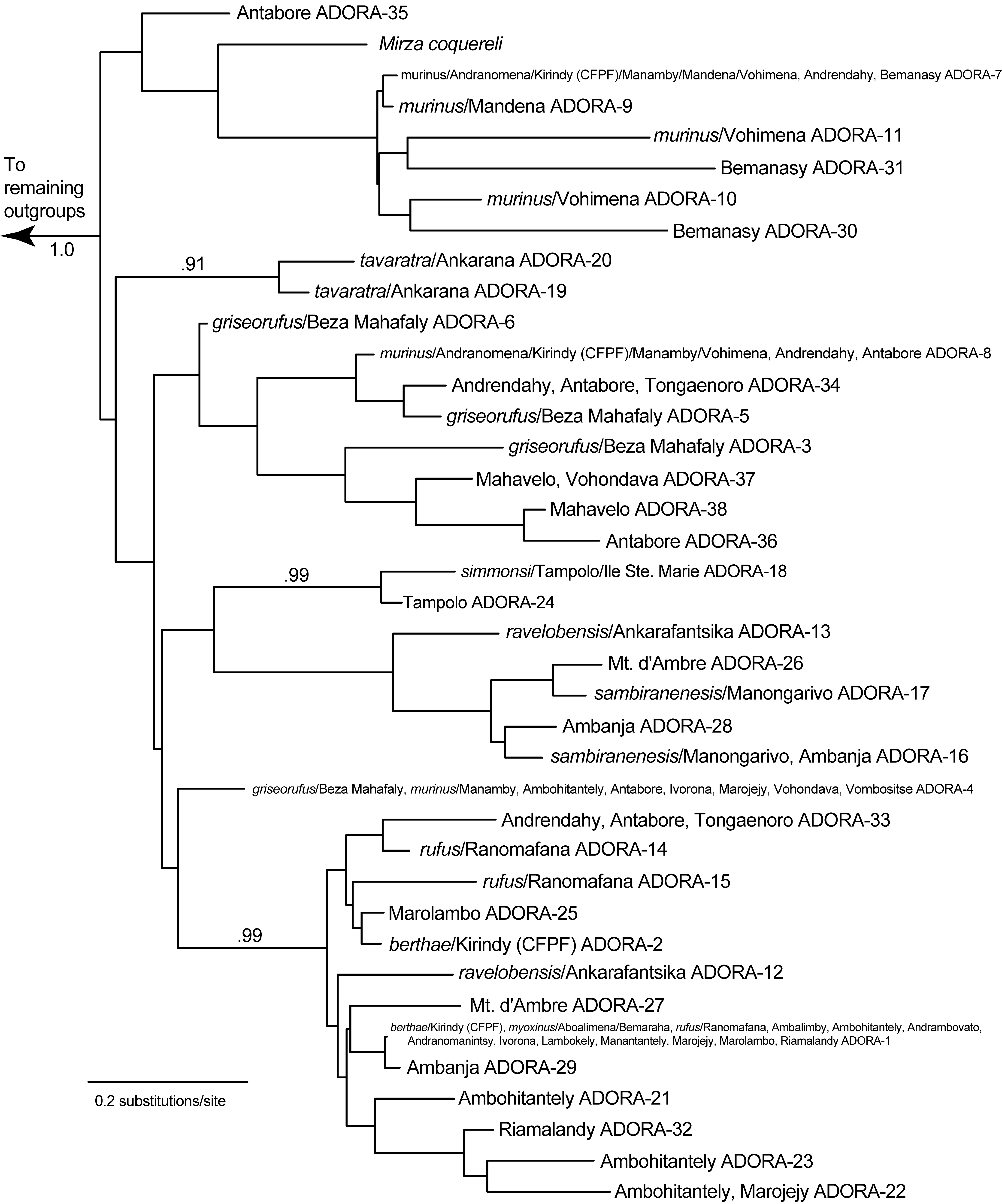

Supplement: Figure S2 — adora3 gene tree. The tree results from Bayesian phylogenetic analysis of the adora3 haplotype data set and is presented as the maximum credible topology with branch lengths averaged across the posterior distribution (Mean -lnL = 1104.67, 95% HPD = 1118.35-1093.08). Tip labels include a species name if the haplotype was sampled from an individual identified to a species in a previous study. Haplotypes recovered from newly sampled individuals are indicated with the locality name. (1.54 MB TIF) [file pone.0009883.s004.tif]

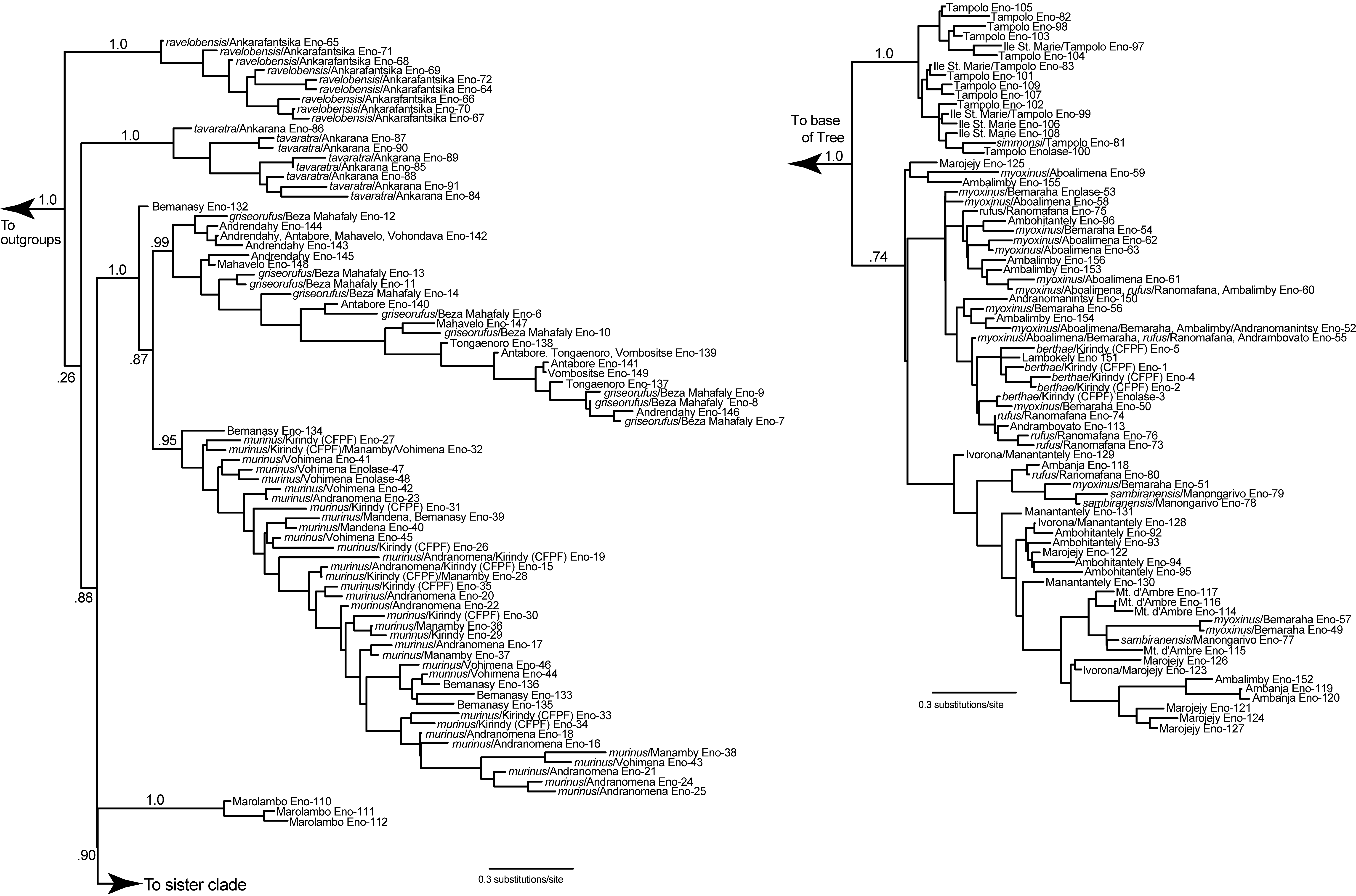

Supplement: Figure S3 — eno gene tree. The tree results from Bayesian phylogenetic analysis of the eno haplotype data set and is presented as the maximum credible topology with branch lengths averaged across the posterior distribution (Mean -lnL = 4557.6, 95%HPD = 4582.15-4531.58). Tip labels include a species name if the haplotype was sampled from an individual identified to a species in a previous study. Haplotypes recovered from newly sampled individuals are indicated with the locality name. (1.74 MB TIF) [file pone.0009883.s005.tif]

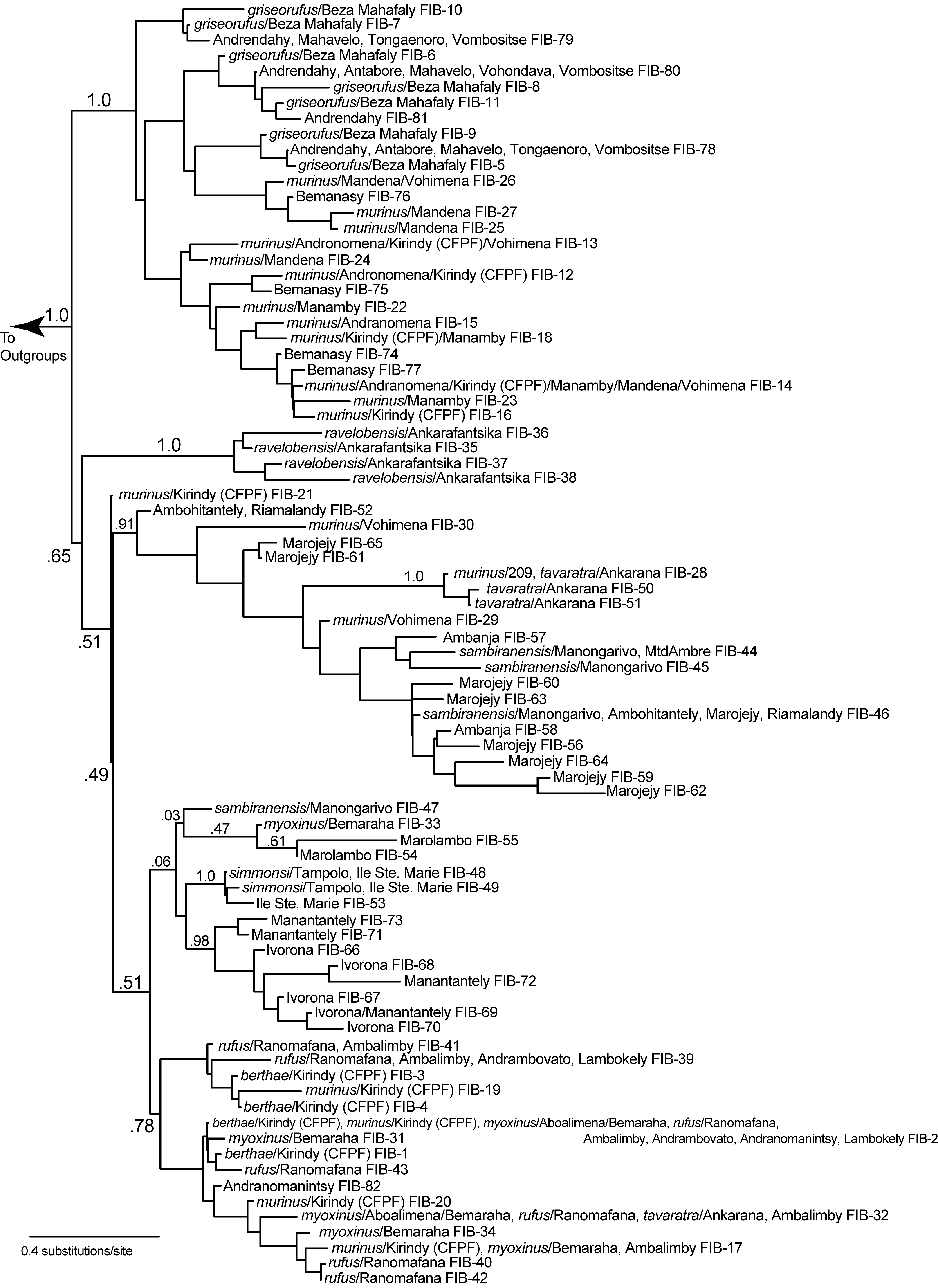

Supplement: Figure S4 — fga gene tree. The tree results from Bayesian phylogenetic analysis of the fga haplotype data set and is presented as the maximum credible topology with branch lengths averaged across the posterior distribution (Mean -lnL = 2232.92, 95%HPD = 2251.07-2215.76). Tip labels include a species name if the haplotype was sampled from an individual identified to a species in a previous study. Haplotypes recovered from newly sampled individuals are indicated with the locality name. (1.84 MB TIF) [file pone.0009883.s006.tif]

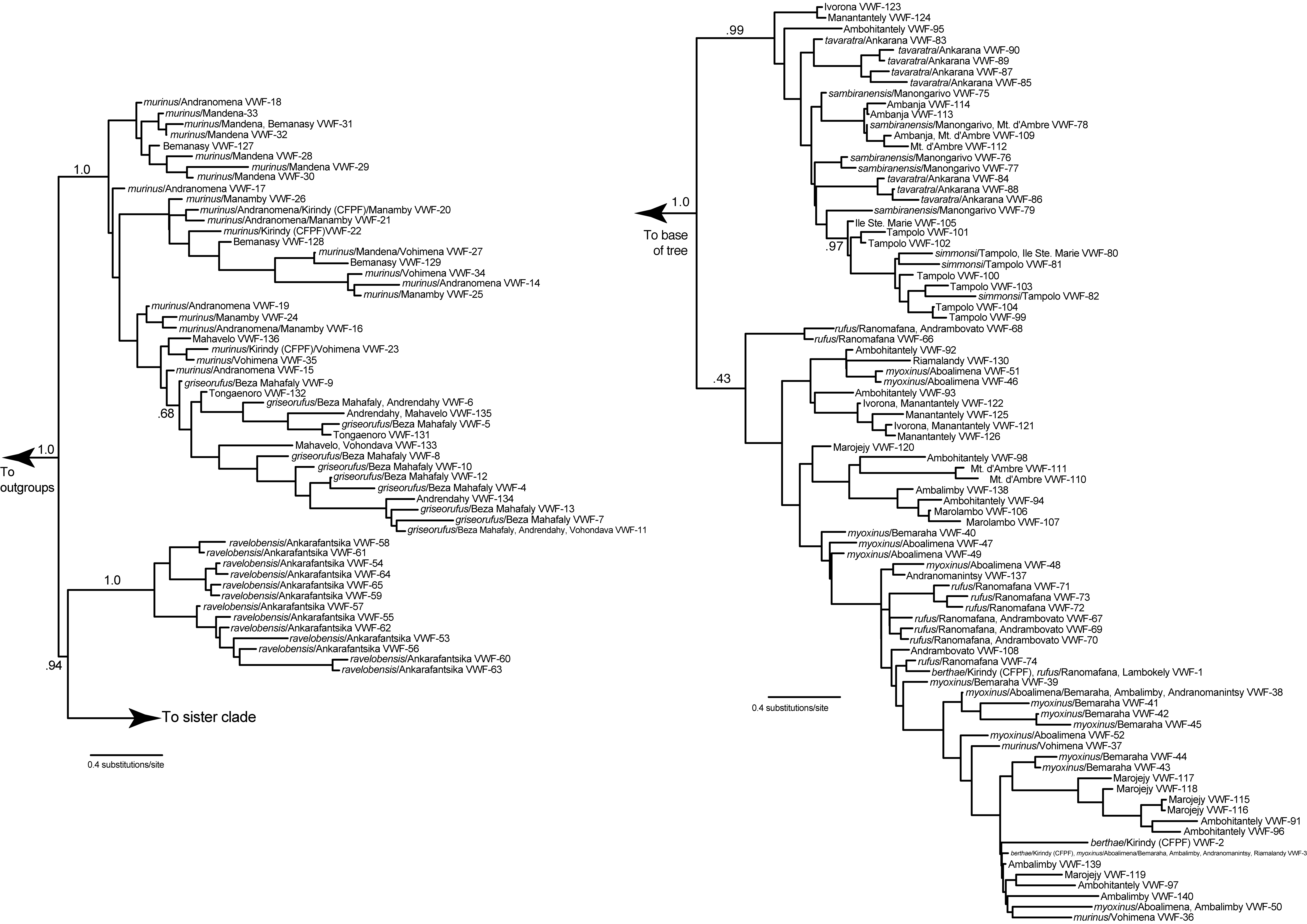

Supplement: Figure S5 — vwf gene tree. The tree results from Bayesian phylogenetic analysis of the vwf haplotype data set and is presented as the maximum credible topology with branch lengths averaged across the posterior distribution (Mean -lnL = 3980.14, 95%HPD = 4007.55-3953.37). Tip labels include a species name if the haplotype was sampled from an individual identified to a species in a previous study. Haplotypes recovered from newly sampled individuals are indicated with the locality name. (1.81 MB TIF) [file pone.0009883.s007.tif]

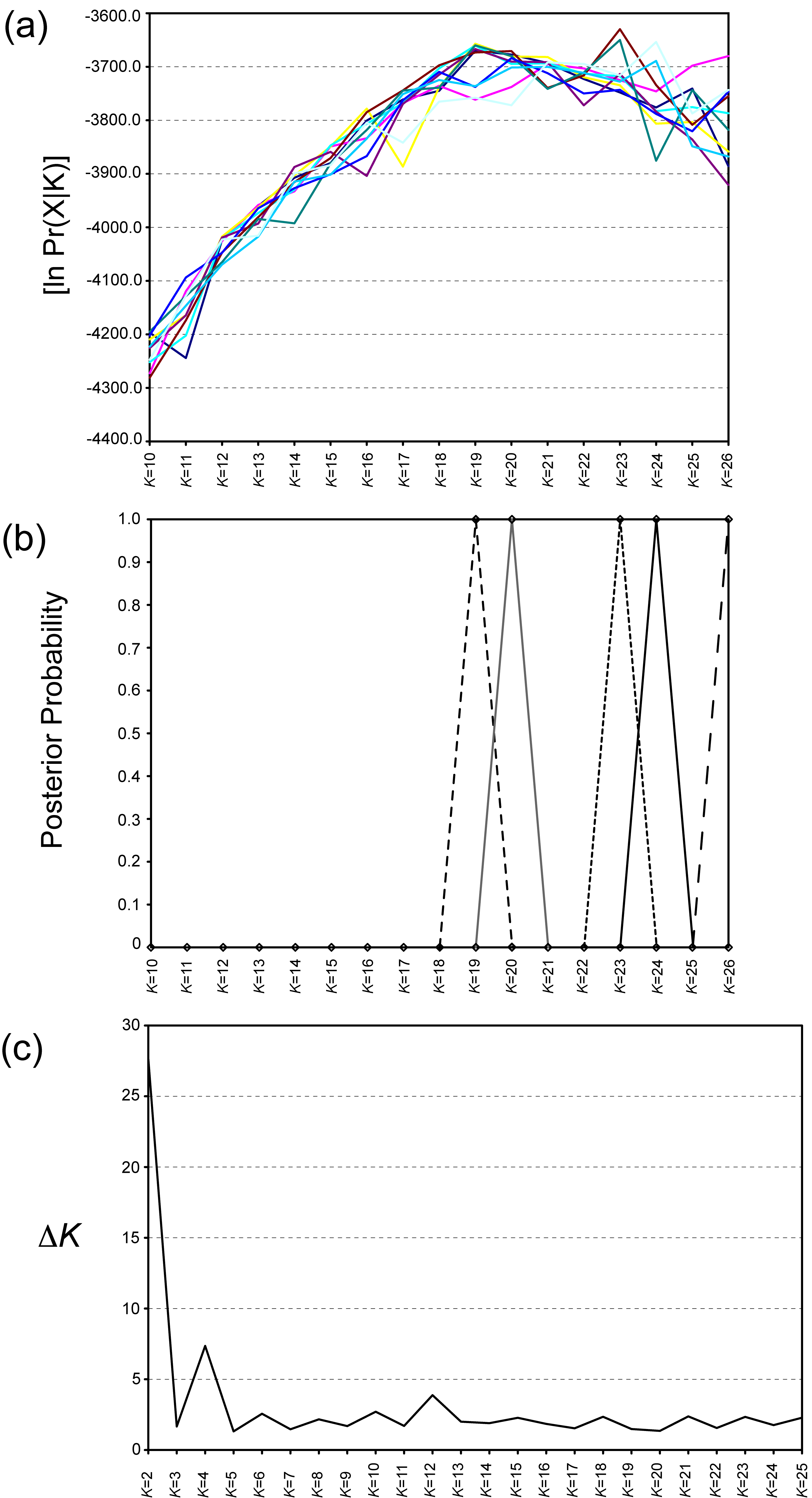

Supplement: Figure S6 — Plots of calculations for various K values in STRUCTURE analysis of the nuclear data. (a) The log probability of the data for K = 10 to 26. Colored lines represent replicate STRUCTURE analyses. (b) Posterior probabilities for K = 10 to 26 for replicate STRUCTURE analyses. Different dashed lines represent replicate analyses, many of which have the same posterior probability. (c) ΔK values for K = 2 to 25. (1.55 MB TIF) [file pone.0009883.s008.tif]
